# Supplementary material for: Multilayer regulation underlies the functional precision and evolutionary potential of the olfactory system
Source: Nat Commun. 2025 Oct 28;16:9514. doi: 10.1038/s41467-025-64514-8 (PMC12569201; doi:10.1038/s41467-025-64514-8)
Supplement: Supplementary file 1 — Supplementary Information [file 41467_2025_64514_MOESM1_ESM.pdf]

Supplementary Information

Multilayer regulation underlies the functional precision and evolutionary potential of the olfactory system

Jérôme Mermet, Steeve Cruchet, Asfa Sabrin Borbora, Daehan Lee,
Phing Chian Chai, Andre Jang, Karen Menuz, Richard Benton

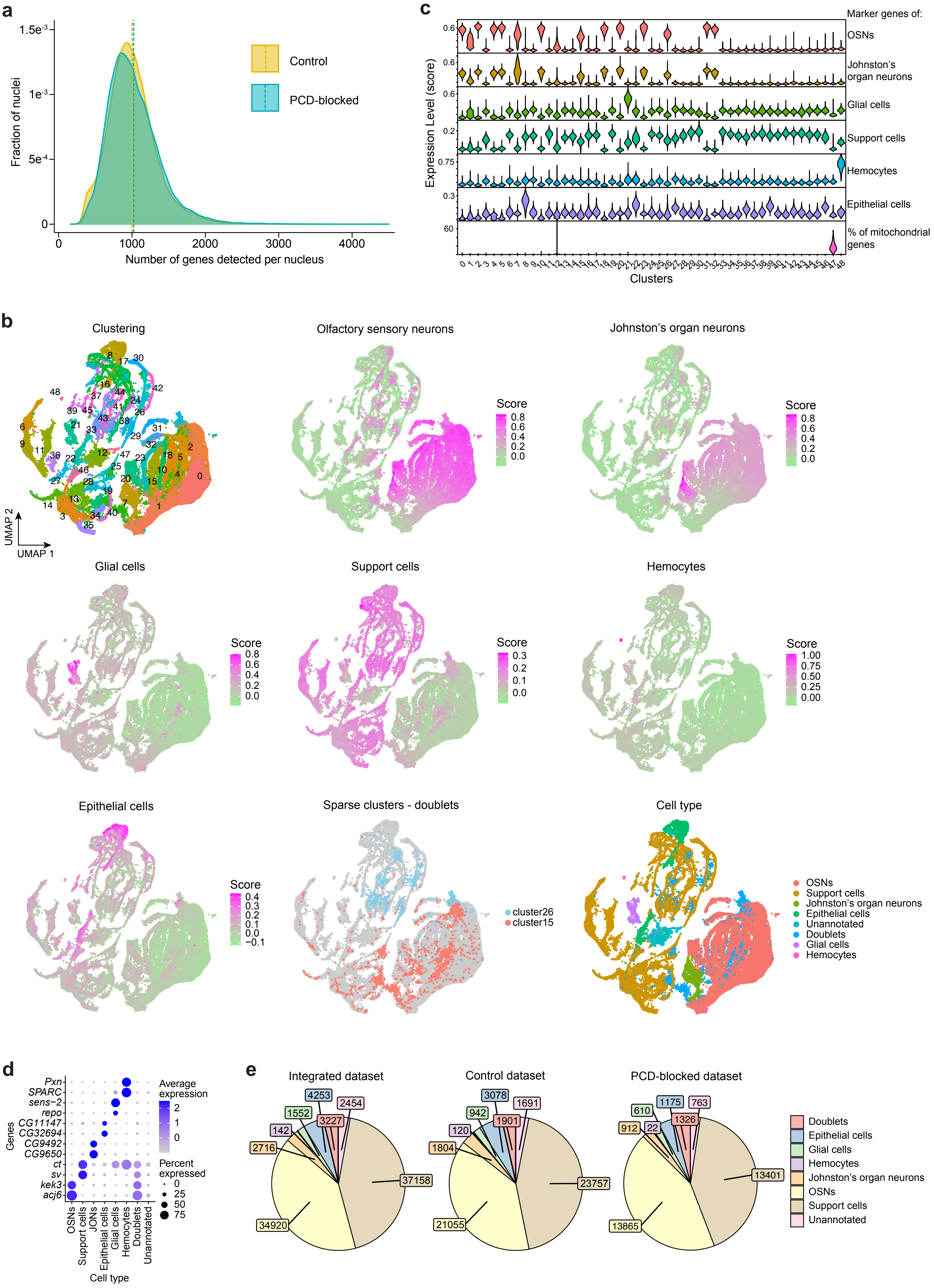
a
Fraction of nuclei
1.5e-3
1e-3
5e-4
0
Control
PCD-blocked
0
1000
2000
3000
4000
Number of genes detected per nucleus
c
Marker genes of:
OSNs
Johnston's organ neurons
Glial cells
Support cells
Hemocytes
Epithelial cells
% of mitochondrial genes
Expression Level (score)
Clusters
b
Clustering
UMAP 2
UMAP 1
Olfactory sensory neurons
Johnston's organ neurons
Score
Glial cells
Support cells
Hemocytes
Epithelial cells
Sparse clusters - doublets
cluster26
cluster15
Cell type
OSNs
Support cells
Johnston's organ neurons
Epithelial cells
Unannotated
Doublets
Glial cells
Hemocytes
d
Pxn
SPARC
sens−2
repo
CG11147
CG32694
CG9492
CG9650
ct
sv
kek3
acj6
Genes
Average expression
Percent expressed
OSNs
Support cells
JONs
Epithelial cells
Glial cells
Hemocytes
Doublets
Unannotated
Cell type
e
Integrated dataset
Control dataset
PCD-blocked dataset
4253
2454
1552
3227
142
2716
37158
34920
3078
1691
942
1901
120
1804
23757
21055
610
1175
763
912
22
1326
13401
13865
Doublets
Epithelial cells
Glial cells
Hemocytes
Johnston's organ neurons
OSNs
Support cells
Unannotated

Supplementary Fig. 1. Global cell type annotation of the developing antenna.
a Distribution of the number of genes detected per nucleus across all sequenced nuclei in control and PCD-blocked datasets. The dashed vertical lines indicate the mean.
b UMAPs illustrating the unsupervised clustering of all nuclei from the integrated control and PCD-blocked datasets (top left), cell type scoring using marker gene modules extracted from the Fly Cell Atlas[1] (see Methods), and cell type annotation of the integrated datasets (bottom right). Nuclei from clusters 15 and 26 were sparse and exhibited a mixed identity; we therefore assigned these as doublets and discarded them from downstream analyses.
c Expression score of cell type marker gene modules and the fraction of detected mitochondrial genes in each cluster.
d Expression pattern of two marker genes per cell type (JON = Johnston's organ neurons) extracted from[1-3] in the annotated control and PCD-blocked integrated datasets, validating cell type assignment.
e Cell type and nuclei number composing the integrated, control and PCD-blocked datasets.

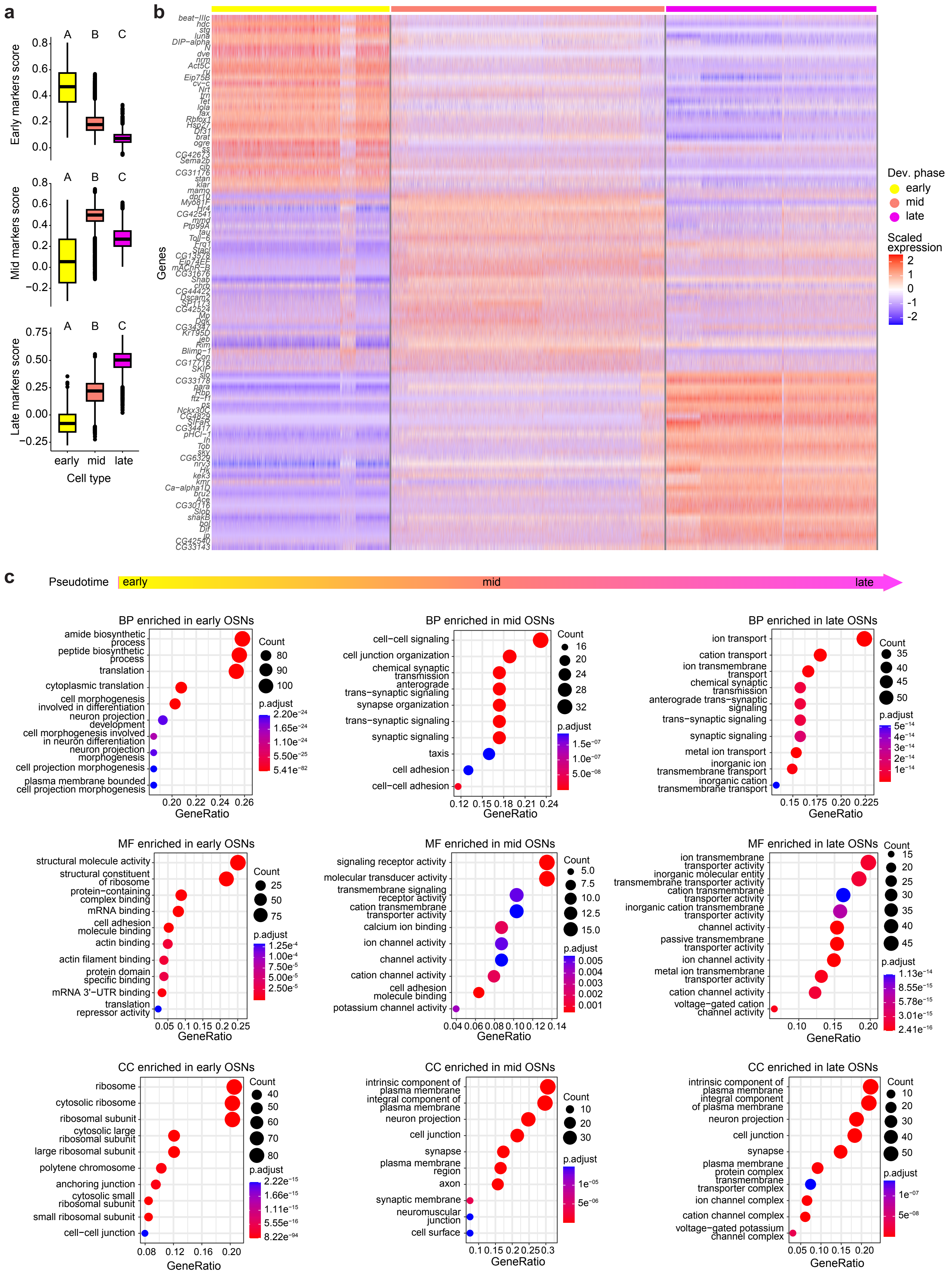
a
b
c
Early markers score
Mid markers score
Late markers score
A
B
C
early
mid
late
Cell type
Genes
Dev. phase
early
mid
late
Scaled expression
Pseudotime
early
mid
late
BP enriched in early OSNs
amide biosynthetic process
peptide biosynthetic process
translation
cytoplasmic translation
cell morphogenesis involved in differentiation
neuron projection development
cell morphogenesis involved in neuron differentiation
neuron projection morphogenesis
cell projection morphogenesis
plasma membrane bounded cell projection morphogenesis
BP enriched in mid OSNs
cell−cell signaling
cell junction organization
chemical synaptic transmission
anterograde trans−synaptic signaling
synapse organization
trans−synaptic signaling
synaptic signaling
taxis
cell adhesion
cell−cell adhesion
BP enriched in late OSNs
ion transport
cation transport
ion transmembrane transport
chemical synaptic transmission
anterograde trans−synaptic signaling
trans−synaptic signaling
synaptic signaling
metal ion transport
inorganic ion transmembrane transport
inorganic cation transmembrane transport
MF enriched in early OSNs
structural molecule activity
structural constituent of ribosome
protein−containing complex binding
mRNA binding
cell adhesion molecule binding
actin binding
actin filament binding
protein domain specific binding
mRNA 3'−UTR binding
translation repressor activity
MF enriched in mid OSNs
signaling receptor activity
molecular transducer activity
transmembrane signaling receptor activity
cation transmembrane transporter activity
calcium ion binding
ion channel activity
channel activity
cation channel activity
cell adhesion molecule binding
potassium channel activity
MF enriched in late OSNs
ion transmembrane transporter activity
inorganic molecular entity transmembrane transporter activity
cation transmembrane transporter activity
inorganic cation transmembrane transporter activity
channel activity
passive transmembrane transporter activity
ion channel activity
metal ion transmembrane transporter activity
cation channel activity
voltage−gated cation channel activity
CC enriched in early OSNs
ribosome
cytosolic ribosome
ribosomal subunit
cytosolic large ribosomal subunit
large ribosomal subunit
polytene chromosome
anchoring junction
cytosolic small ribosomal subunit
small ribosomal subunit
cell−cell junction
CC enriched in mid OSNs
intrinsic component of plasma membrane
integral component of plasma membrane
neuron projection
cell junction
synapse
plasma membrane region
axon
synaptic membrane
neuromuscular junction
cell surface
CC enriched in late OSNs
intrinsic component of plasma membrane
integral component of plasma membrane
neuron projection
cell junction
synapse
plasma membrane protein complex
transmembrane transporter complex
ion channel complex
cation channel complex
voltage−gated potassium channel complex
Count
p.adjust
GeneRatio

Supplementary Fig. 2. Hallmarks of peripheral olfactory system development.
a Expression score of early, mid and late developmental phase gene modules in early, mid and late antennal sensory neurons (integrated datasets). Boxes show the median (thick line), first and third quartiles, while whiskers indicate data distribution limits. Letters indicate significant differences: $P < 0.05$ in pairwise comparisons (Wilcoxon rank sum test followed by Bonferroni correction for multiple comparisons).
b Expression of the top 30 genes (log_2FC) of each developmental time marker module in neurons grouped by developmental time (integrated datasets).
c Gene Ontology (GO) analysis illustrating the top 10 (log_{10}(adjusted P)) Biological Process (BP), Molecular Function (MF) and Cellular Component (CC) categories enriched in early, mid and late developmental phase gene modules (810 genes total).

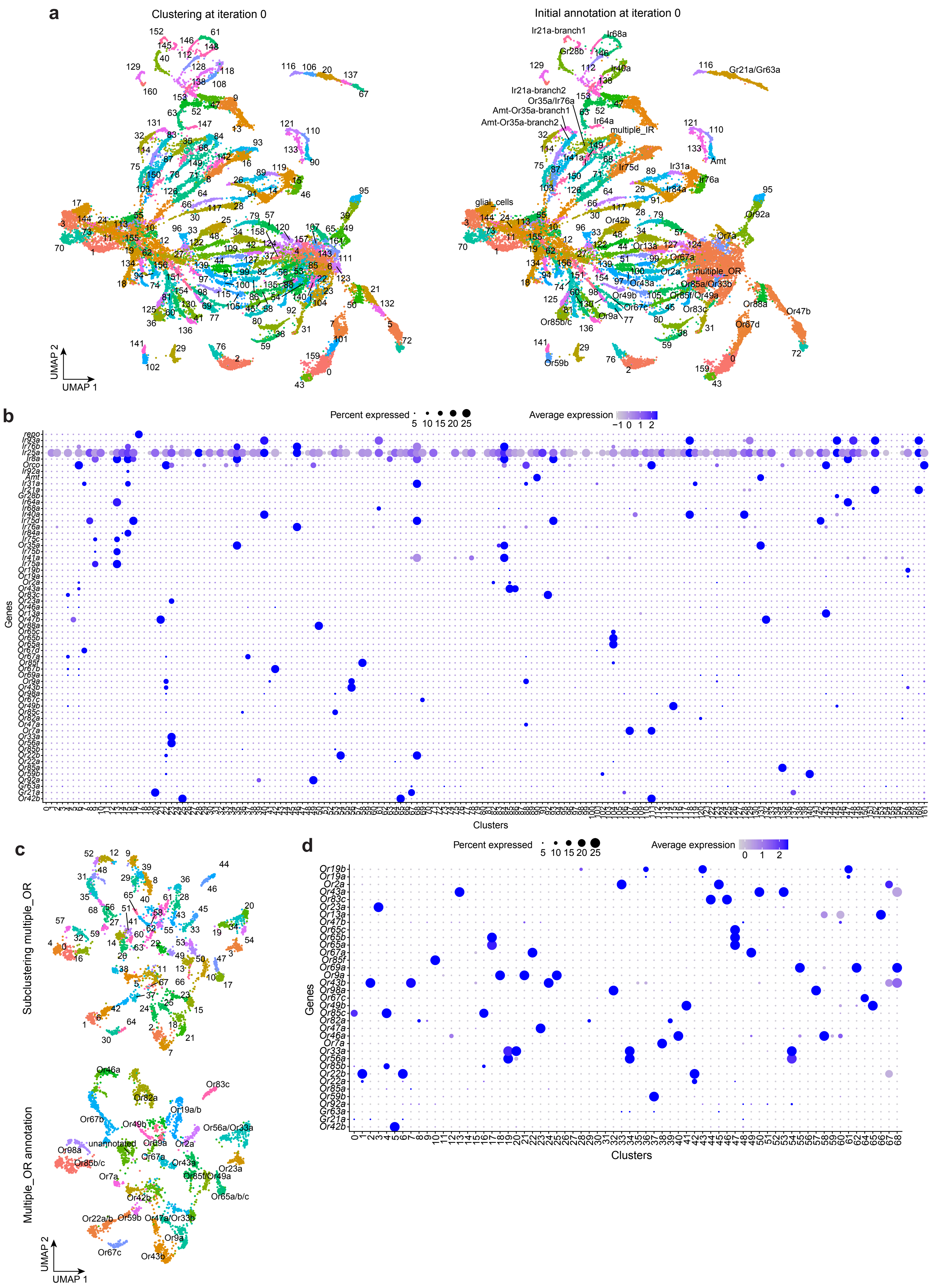
a
Clustering at iteration 0
Initial annotation at iteration 0
Ir21a-branch1
Ir68a
Gr28b
Ir40a
Gr21a/Gr63a
Ir21a-branch2
Or35a/Ir76a
Amt-Or35a-branch1
Amt-Or35a-branch2
Ir64a
multiple_IR
Ir41a
Ir75d
Ir31a
Ir76a
Ir84a
Amt
glial_cells
Or42b
Or92a
Or7a
Or13a
Or67a
Or2a
multiple_OR
Or43a
Or85a/Or33b
Or49b
Or85f/Or49a
Or67c
Or83c
Or88a
Or47b
Or9a
Or85b/c
Or67d
Or59b
UMAP 2
UMAP 1
b
Percent expressed
5 10 15 20 25
Average expression
−1 0 1 2
Genes
Clusters
c
Subclustering multiple_OR
Multiple_OR annotation
Or46a
Or83c
Or82a
Or19a/b
Or67b
Or49b
Or56a/Or33a
Or98a
unannotated
Or69a
Or2a
Or67a
Or85b/c
Or43a
Or23a
Or7a
Or85f/Or49a
Or65a/b/c
Or42b
Or22a/b
Or59b
Or47a/Or33b
Or9a
Or67c
Or43b
UMAP 2
UMAP 1
d
Percent expressed
5 10 15 20 25
Average expression
0 1 2
Genes
Clusters

Supplementary Fig. 3. Initial annotation of neuronal subclusters based upon sensory receptor expression.

a UMAPs of unsupervised clustering of the sensory neurons (integrated datasets) at iteration 0 (left) and the initial annotation of a subset of these (typically late-stage cells) based upon sensory receptor gene expression in the control dataset (right).

b Expression of diagnostic receptor and co-receptor subunit genes of both the Or and Ir subsystem (and the glial marker *repo*) in each cluster from **a** (left UMAP, control dataset only).

c UMAPs of unsupervised sub-clustering of the multiple_OR cluster from **a** (right UMAP) (top) and the annotation based upon sensory receptor gene expression in the control dataset (bottom).

d Expression of diagnostic *Or* and *Ir* genes in each cluster in **c** (top UMAP; control dataset only).

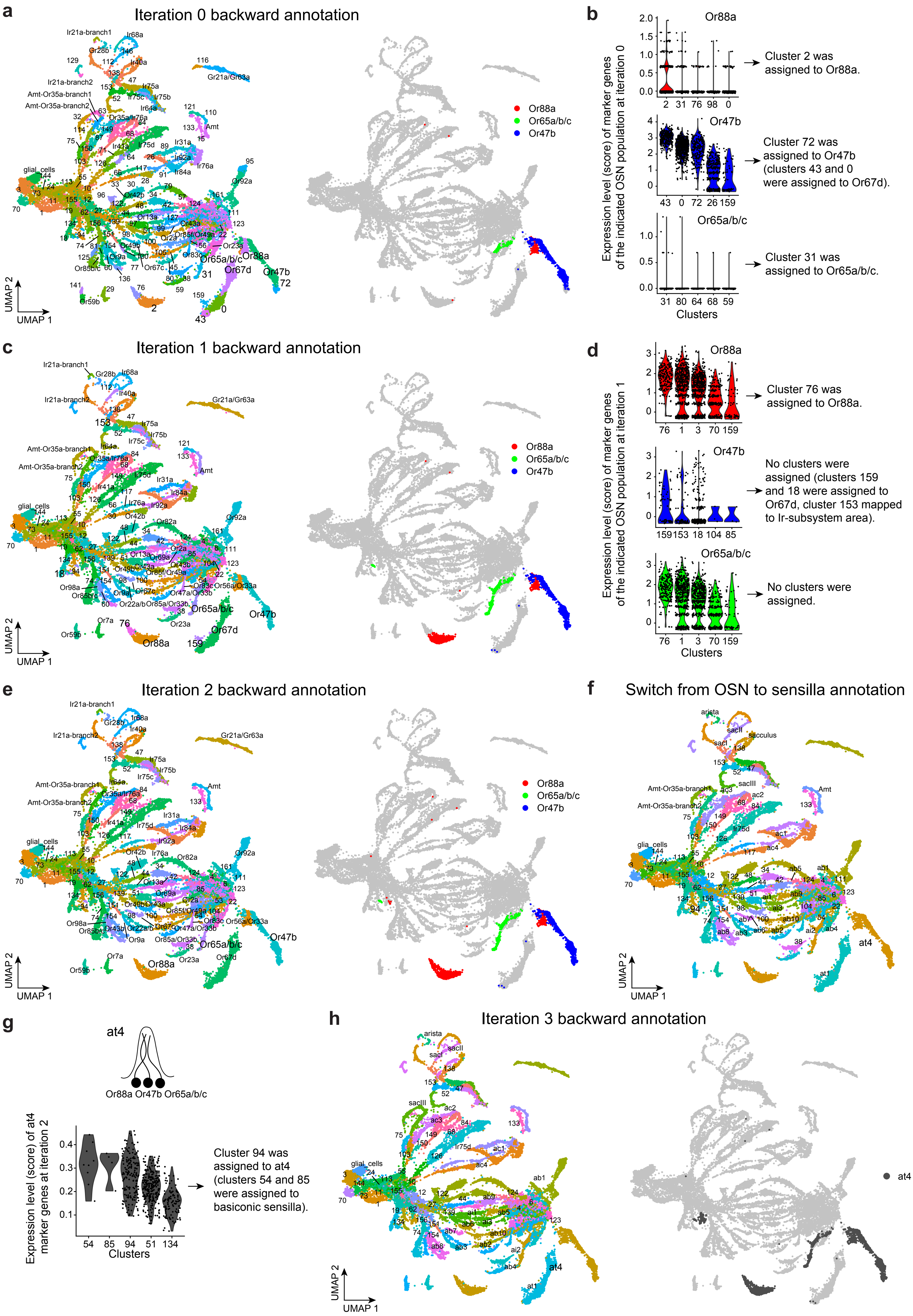

a
Iteration 0 backward annotation
Or88a
Or65a/b/c
Or47b
UMAP 2
UMAP 1
b
Expression level (score) of marker genes of the indicated OSN population at iteration 0
Cluster 2 was assigned to Or88a.
Cluster 72 was assigned to Or47b (clusters 43 and 0 were assigned to Or67d).
Cluster 31 was assigned to Or65a/b/c.
Clusters
c
Iteration 1 backward annotation
d
Expression level (score) of marker genes of the indicated OSN population at iteration 1
Cluster 76 was assigned to Or88a.
No clusters were assigned (clusters 159 and 18 were assigned to Or67d, cluster 153 mapped to Ir-subsystem area).
No clusters were assigned.
e
Iteration 2 backward annotation
f
Switch from OSN to sensilla annotation
g
at4
Or88a Or47b Or65a/b/c
Expression level (score) of at4 marker genes at iteration 2
Clusters
Cluster 94 was assigned to at4 (clusters 54 and 85 were assigned to basiconic sensilla).
h
Iteration 3 backward annotation
at4

Supplementary Fig. 4. Backward, iterative annotation of subclusters based upon sensory receptor expression.

a UMAPs at iteration 0 of sensory neuron annotations (left) and highlighting at4 neurons Or88a (red), Or65a/b/c (green) and Or47b (blue) (right).

b Ranked expression score (left-to-right) of each at4 OSN marker gene module extracted from the iteration 0 annotated dataset. The top 5 unannotated clusters (i.e., excluding the clusters already annotated based upon receptor expression) with the highest scores for each OSN population marker genes modules are shown; these scores allowed us to assign clusters from earlier developmental phase (typically lacking sensory receptor expression) to each at4 OSN, as indicated in the text on the right. These clusters were used for a subsequent round of marker gene scoring. Where several clusters had a similar marker gene score, we incorporated information from selected marker genes and the relative position of clusters within the UMAP to assign identity; here, for example, Or47b neuron marker genes have a high score in 3 clusters (43, 0, 72), but as Or67d neuron marker genes also have a high score in clusters 43 and 0 at the same iteration and these two clusters form a continuum with the Or67d cluster, they were ultimately assigned to the Or67d lineage and cluster 72 (adjacent to the Or47b cluster at iteration 0) was assigned to the Or47b lineage.

c UMAPs at iteration 1 of sensory neuron annotations (left) and highlighting Or88a (red), Or65a/b/c (green) and Or47b (blue) neurons (right).

d Ranked expression score (left-to-right) of each at4 OSN marker gene module extracted from the iteration 1 annotated object. The top 5 unannotated clusters with the highest scores for each OSN population marker gene modules are shown, allowing us to assign clusters to Or88a but not Or47b or Or65a/b/c OSNs, as indicated in the text on the right). These clusters were used for a subsequent round of marker gene scoring.

e UMAPs at iteration 2 of sensory neurons (left) and highlighting Or88a (red), Or65/a/b/c (green) and Or47b (blue) neurons (right). No further sensory neuron lineage-based backward annotation was possible.

f UMAP at iteration 2 (as in **e**) with sensilla-based annotations.

g Ranked expression score (left-to-right) of the at4 sensillum marker gene module extracted from the iteration 2, sensilla-based, annotated object. The top 5 unannotated clusters with the highest scores are shown, allowing us to assign clusters to at4, as indicated in the text on the right. These clusters were used for a subsequent round of marker gene scoring.

h UMAPs at iteration 3 of the sensilla-based annotation of sensory neurons (left) and highlighting the at4 sensillum (right).

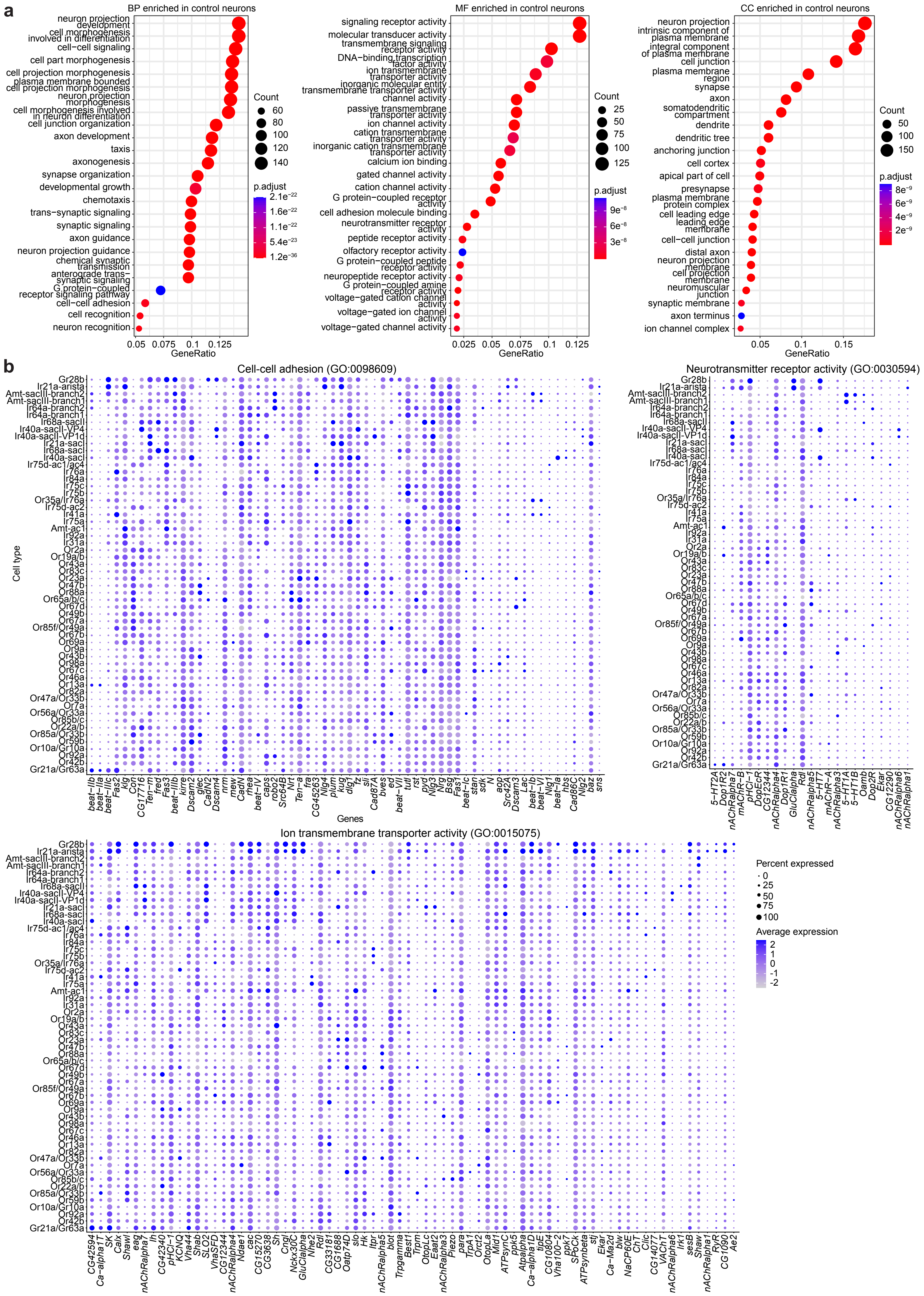

a
BP enriched in control neurons
neuron projection development
cell morphogenesis involved in differentiation
cell−cell signaling
cell part morphogenesis
cell projection morphogenesis
plasma membrane bounded cell projection morphogenesis
neuron projection morphogenesis
cell morphogenesis involved in neuron differentiation
cell junction organization
axon development
taxis
axonogenesis
synapse organization
developmental growth
chemotaxis
trans−synaptic signaling
synaptic signaling
axon guidance
neuron projection guidance
chemical synaptic transmission
anterograde trans−synaptic signaling
G protein−coupled receptor signaling pathway
cell−cell adhesion
cell recognition
neuron recognition
GeneRatio
Count
p.adjust
MF enriched in control neurons
signaling receptor activity
molecular transducer activity
transmembrane signaling receptor activity
DNA−binding transcription factor activity
ion transmembrane transporter activity
inorganic molecular entity transmembrane transporter activity
channel activity
passive transmembrane transporter activity
ion channel activity
cation transmembrane transporter activity
inorganic cation transmembrane transporter activity
calcium ion binding
gated channel activity
cation channel activity
G protein−coupled receptor activity
cell adhesion molecule binding
neurotransmitter receptor activity
peptide receptor activity
olfactory receptor activity
G protein−coupled peptide receptor activity
neuropeptide receptor activity
G protein−coupled amine receptor activity
voltage−gated cation channel activity
voltage−gated ion channel activity
voltage−gated channel activity
CC enriched in control neurons
neuron projection
intrinsic component of plasma membrane
integral component of plasma membrane
cell junction
plasma membrane region
synapse
axon
somatodendritic compartment
dendrite
dendritic tree
anchoring junction
cell cortex
apical part of cell
presynapse
plasma membrane protein complex
cell leading edge
leading edge membrane
cell−cell junction
distal axon
neuron projection membrane
cell projection membrane
neuromuscular junction
synaptic membrane
axon terminus
ion channel complex
b
Cell-cell adhesion (GO:0098609)
Neurotransmitter receptor activity (GO:0030594)
Ion transmembrane transporter activity (GO:0015075)
Cell type
Genes
Percent expressed
Average expression

Supplementary Fig. 5. Sensory neuron type marker genes.
a GO analysis illustrating the top 25 (log_{10}(adjusted P)) Biological Process (BP), Molecular Function (MF) and Cellular Component (CC) categories enriched in OSNs marker gene modules (1242 genes total). Only the control dataset was analyzed in this and the following panel.
b Expression of OSN marker genes belonging to the indicated GO categories.

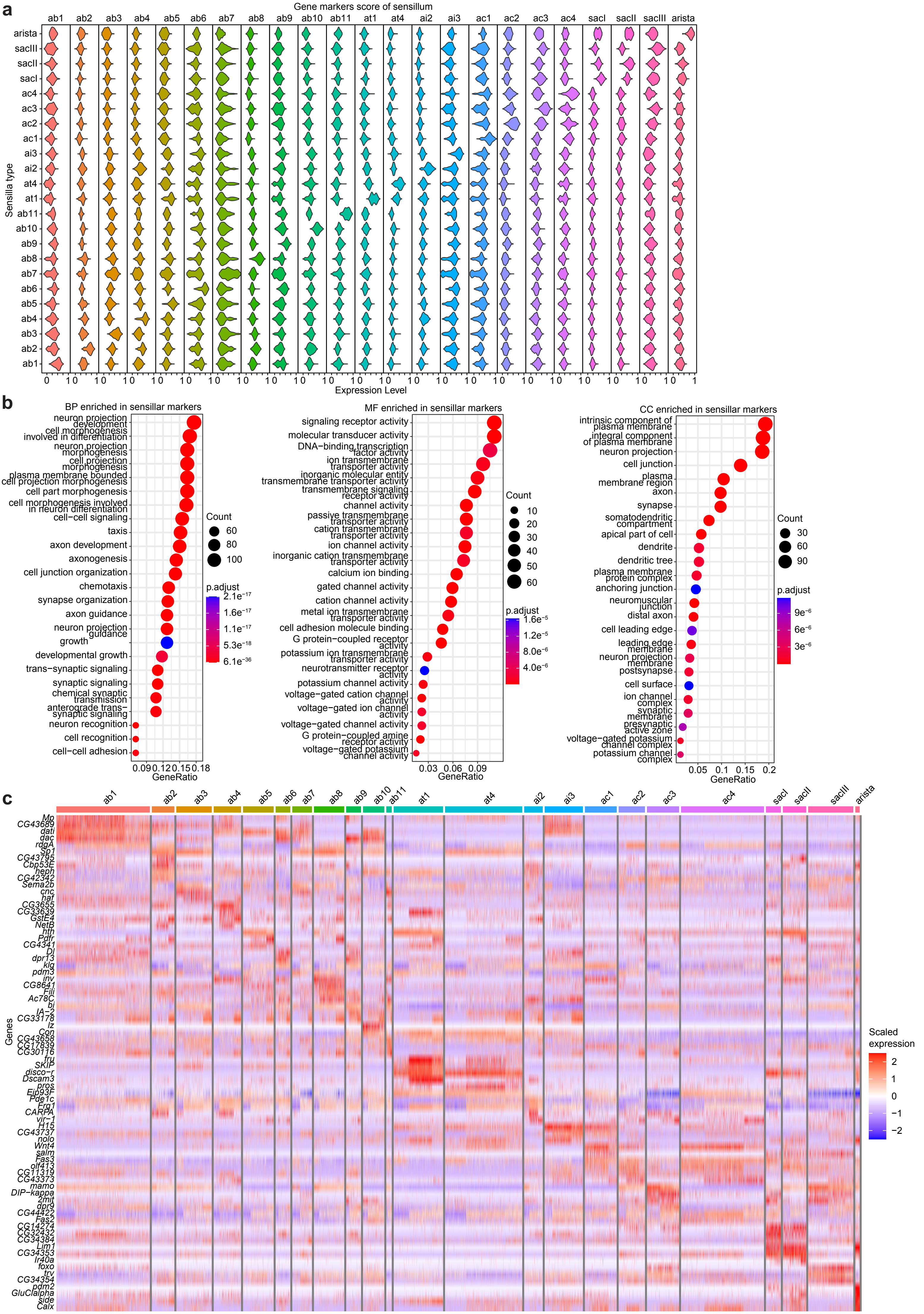
a
Gene markers score of sensillum
ab1 ab2 ab3 ab4 ab5 ab6 ab7 ab8 ab9 ab10 ab11 at1 at4 ai2 ai3 ac1 ac2 ac3 ac4 sacI sacII sacIII arista
Sensilla type
arista
sacIII
sacII
sacI
ac4
ac3
ac2
ac1
ai3
ai2
at4
at1
ab11
ab10
ab9
ab8
ab7
ab6
ab5
ab4
ab3
ab2
ab1
Expression Level
b
BP enriched in sensillar markers
neuron projection development
cell morphogenesis involved in differentiation
neuron projection morphogenesis
cell projection morphogenesis
plasma membrane bounded cell projection morphogenesis
cell part morphogenesis
cell morphogenesis involved in neuron differentiation
cell−cell signaling
taxis
axon development
axonogenesis
cell junction organization
chemotaxis
synapse organization
axon guidance
neuron projection guidance
growth
developmental growth
trans−synaptic signaling
synaptic signaling
chemical synaptic transmission
anterograde trans−synaptic signaling
neuron recognition
cell recognition
cell−cell adhesion
GeneRatio
0.09 0.12 0.15 0.18
Count
60
80
100
p.adjust
2.1e−17
1.6e−17
1.1e−17
5.3e−18
6.1e−36
MF enriched in sensillar markers
signaling receptor activity
molecular transducer activity
DNA−binding transcription factor activity
ion transmembrane transporter activity
inorganic molecular entity transmembrane transporter activity
transmembrane signaling receptor activity
channel activity
passive transmembrane transporter activity
cation transmembrane transporter activity
ion channel activity
inorganic cation transmembrane transporter activity
calcium ion binding
gated channel activity
cation channel activity
metal ion transmembrane transporter activity
cell adhesion molecule binding
G protein−coupled receptor activity
potassium ion transmembrane transporter activity
neurotransmitter receptor activity
potassium channel activity
voltage−gated cation channel activity
voltage−gated ion channel activity
voltage−gated channel activity
G protein−coupled amine receptor activity
voltage−gated potassium channel activity
GeneRatio
0.03 0.06 0.09
Count
10
20
30
40
50
60
p.adjust
1.6e−5
1.2e−5
8.0e−6
4.0e−6
CC enriched in sensillar markers
intrinsic component of plasma membrane
integral component of plasma membrane
neuron projection
cell junction
plasma membrane region
axon
synapse
somatodendritic compartment
apical part of cell
dendrite
dendritic tree
plasma membrane protein complex
anchoring junction
neuromuscular junction
distal axon
cell leading edge
leading edge membrane
neuron projection membrane
postsynapse
cell surface
ion channel complex
synaptic membrane
presynaptic active zone
voltage−gated potassium channel complex
potassium channel complex
GeneRatio
0.05 0.1 0.15 0.2
Count
30
60
90
p.adjust
9e−6
6e−6
3e−6
c
ab1 ab2 ab3 ab4 ab5 ab6 ab7 ab8 ab9 ab10 ab11 at1 at4 ai2 ai3 ac1 ac2 ac3 ac4 sacI sacII sacIII arista
Genes
Mp
CG43689
dati
dac
rdgA
Sp1
CG43795
Cbp53E
heph
CG42342
Sema2b
cnc
haf
CG3655
CG33639
GstE4
NetB
hth
Pdfr
CG4341
Dl
dpr13
klg
pdm3
inv
CG8641
Fili
Ac78C
bi
IA−2
CG33178
lz
Con
CG43658
CG17839
CG30116
fru
SKIP
disco−r
Dscam3
pros
Eip93F
Pde1c
Fz4
CARPA
vir−1
H15
CG43737
nolo
Wnt4
salm
Fas3
olf413
CG11319
CG43373
mamo
DIP−kappa
2mit
dpr9
CG44422
Fas2
CG14274
CG32432
CG34384
Lim1
CG34353
Ir40a
foxo
trv
CG34354
pdm2
GluClalpha
side
Calx
Scaled expression
2
1
0
−1
−2

Supplementary Fig. 6. Sensilla marker genes.

a Expression score of sensilla marker gene modules across sensilla (control dataset only in this and other panels).

b Gene Ontology (GO) analysis illustrating the top 25 (log_{10}(adjusted P)) Biological Process (BP), Molecular Function (MF) and Cellular Component (CC) categories enriched in sensilla marker gene modules (727 genes total).

c Expression of the top 5 marker genes (log_2FC) of each sensillum class.

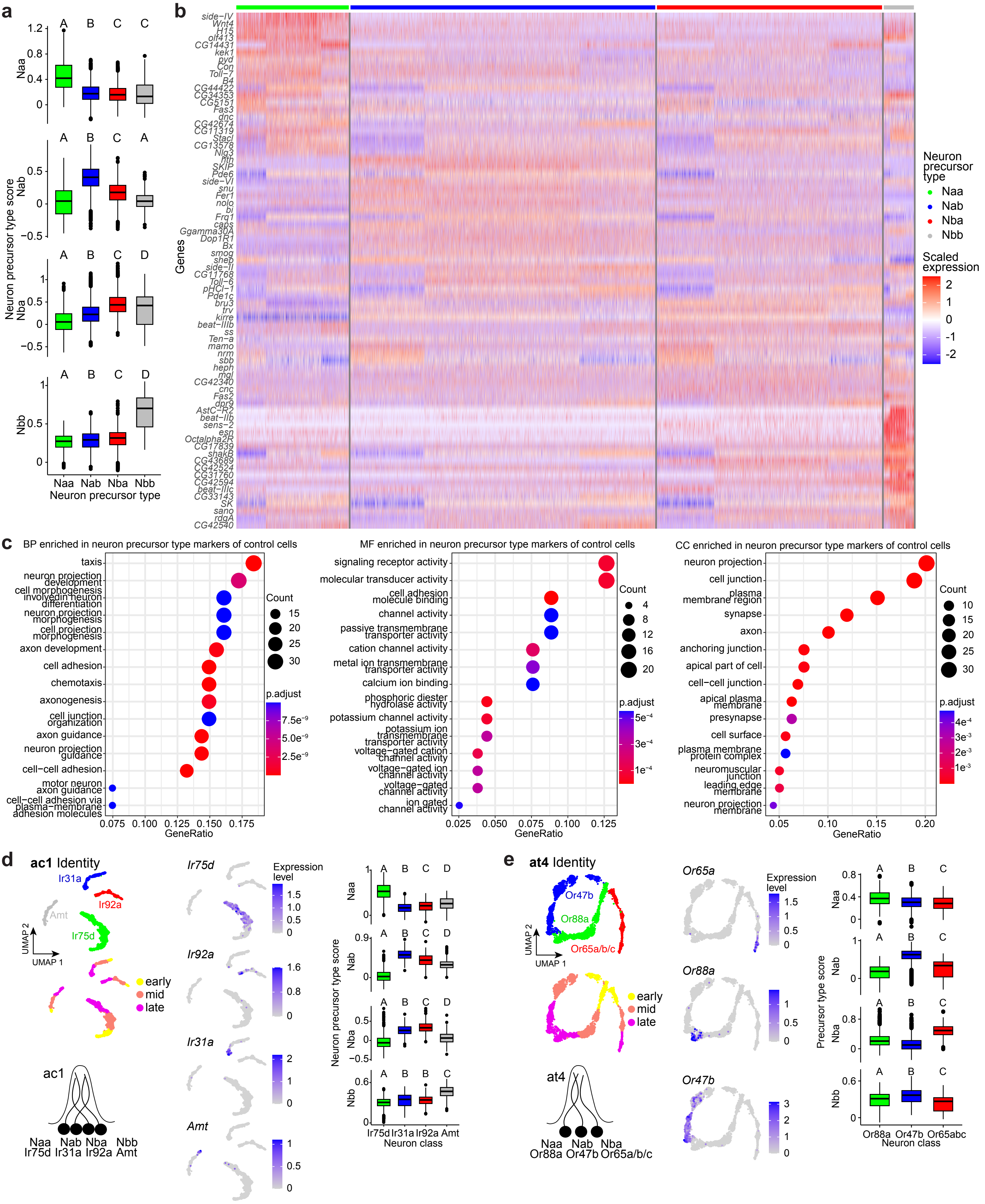
a
b
Neuron precursor type score
Naa
Nab
Nba
Nbb
Neuron precursor type
Genes
side-IV
Wnt4
H15
olf413
CG14431
kek1
pyd
Con
Toll-7
B4
CG44422
CG34353
CG5151
Fas3
dnc
CG42674
CG11319
Stacl
CG13578
Nlg3
fru
SKIP
Pde6
side-VI
snu
Fer1
nolo
bi
Frq1
caps
Ggamma30A
Dop1R1
Bx
smog
shep
side-II
CG11768
Toll-6
pHCl-1
Pde1c
bru3
trv
kirre
beat-IIb
ss
Ten-a
mamo
nrm
sbb
heph
mgl
CG42340
cnc
Fas2
dpr9
AstC-R2
beat-IIb
sens-2
esn
Octalpha2R
CG17839
shakB
CG43689
CG42524
CG31760
CG42594
beat-IIIc
CG33143
SK
sano
rdgA
CG42540
Neuron precursor type
Scaled expression
c
BP enriched in neuron precursor type markers of control cells
taxis
neuron projection development
cell morphogenesis involved in neuron differentiation
neuron projection morphogenesis
cell projection morphogenesis
axon development
cell adhesion
chemotaxis
axonogenesis
cell junction organization
axon guidance
neuron projection guidance
cell-cell adhesion
motor neuron axon guidance
cell-cell adhesion via plasma-membrane adhesion molecules
GeneRatio
Count
p.adjust
MF enriched in neuron precursor type markers of control cells
signaling receptor activity
molecular transducer activity
cell adhesion molecule binding
channel activity
passive transmembrane transporter activity
cation channel activity
metal ion transmembrane transporter activity
calcium ion binding
phosphoric diester hydrolase activity
potassium channel activity
potassium ion transmembrane transporter activity
voltage-gated cation channel activity
voltage-gated ion channel activity
voltage-gated channel activity
ion gated channel activity
CC enriched in neuron precursor type markers of control cells
neuron projection
cell junction
plasma membrane region
synapse
axon
anchoring junction
apical part of cell
cell-cell junction
apical plasma membrane
presynapse
cell surface
plasma membrane protein complex
neuromuscular junction
leading edge membrane
neuron projection membrane
d
ac1 Identity
Ir31a
Ir92a
Amt
Ir75d
UMAP 2
UMAP 1
early
mid
late
ac1
Naa Nab Nba Nbb
Ir75d Ir31a Ir92a Amt
Expression level
Neuron precursor type score
Neuron class
e
at4 Identity
Or47b
Or88a
Or65a/b/c
Or65a
Or88a
Or47b
at4
Naa Nab Nba
Or88a Or47b Or65a/b/c
Precursor type score
Or88a Or47b Or65abc
Neuron class

Supplementary Fig. 7. Sensory neuron precursor type marker genes.
a Expression score of Naa, Nab, Nba and Nbb marker gene modules in each sensory neuron precursor type category. Boxes show the median (thick line), first and third quartiles, while whiskers indicate data distribution limits. Letters indicate significant differences: $P < 0.05$ in pairwise comparisons (Wilcoxon rank sum test followed by Bonferroni correction for multiple comparisons). Only the control dataset was analyzed in this and the following panels.
b Expression of the top 20 genes ($\log_2$FC) from each of the sensory neuron precursor type marker gene modules across sensory neuron precursor types.
c Gene Ontology (GO) analysis showing the top 25 ($\log_{10}$(adjusted P)) Biological Process (BP), Molecular Function (MF) and Cellular Component (CC) categories enriched in sensory neuron precursor type marker gene modules (200 genes total).
d-e UMAPs of ac1 **d** and at4 **e** with annotation and developmental phases (left), the expression of diagnostic sensory receptors (middle), the expression score of sensory neuron precursor type marker gene modules (right) and a schematic illustrating the inferred precursor type and identity of sensory neurons (bottom left). Letters indicate significant differences: $P < 0.05$ in pairwise comparisons (Wilcoxon rank sum test followed by Bonferroni correction for multiple comparisons).

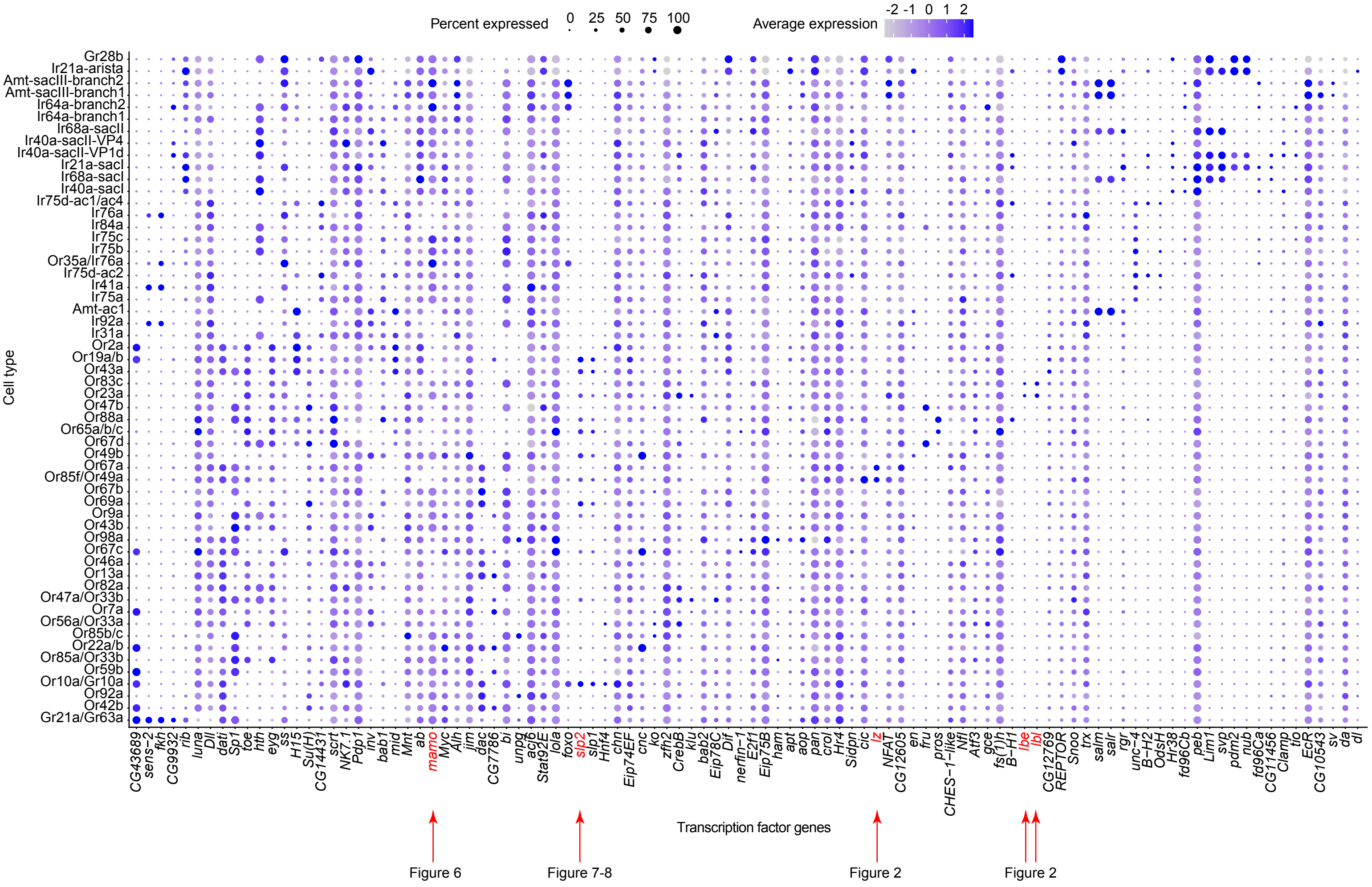

Percent expressed
0
25
50
75
100
Average expression
-2 -1 0 1 2
Cell type
Gr28b
Ir21a-arista
Amt-sacIII-branch2
Amt-sacIII-branch1
Ir64a-branch2
Ir64a-branch1
Ir68a-sacII
Ir40a-sacII-VP4
Ir40a-sacII-VP1d
Ir21a-sacI
Ir68a-sacI
Ir40a-sacI
Ir75d-ac1/ac4
Ir76a
Ir84a
Ir75c
Ir75b
Or35a/Ir76a
Ir75d-ac2
Ir41a
Ir75a
Amt-ac1
Ir92a
Ir31a
Or2a
Or19a/b
Or43a
Or83c
Or23a
Or47b
Or88a
Or65a/b/c
Or67d
Or49b
Or67a
Or85f/Or49a
Or67b
Or69a
Or9a
Or43b
Or98a
Or67c
Or46a
Or13a
Or82a
Or47a/Or33b
Or7a
Or56a/Or33a
Or85b/c
Or22a/b
Or85a/Or33b
Or59b
Or10a/Gr10a
Or92a
Or42b
Gr21a/Gr63a
CG43689
sens−2
fkh
CG9932
rib
luna
Dll
dati
Sp1
toe
hth
eyg
ss
H15
Su(H)
CG14431
scrt
NK7.1
Pdp1
inv
bab1
mid
Mnt
ab
mamo
Myc
Alh
jim
dac
CG7786
bi
unpg
acj6
Stat92E
lola
foxo
slp2
slp1
Hr4
chn
Eip74EF
cnc
ko
zfh2
CrebB
klu
bab2
Eip78C
Dif
nerfin−1
E2f1
Eip75B
ham
apt
aop
pan
crol
Hr4
Sidpn
cic
lz
NFAT
CG12605
en
fru
pros
CHES−1−like
Nfl
Atf3
gce
fs(1)h
B−H1
lbe
lbl
CG12769
REPTOR
Snoo
trx
salm
salr
rgr
unc−4
B−H2
OdsH
Hr38
fd96Cb
peb
Lim1
svp
pdm2
nub
fd96Ca
CG11456
Clamp
tio
EcR
CG10543
sv
da
dl
Transcription factor genes
Figure 6
Figure 7-8
Figure 2
Figure 2

Supplementary Fig. 8. Putative transcription factor codes underlying sensory neuron identity.

Differentially expressed TFs in sensory neuron populations (control dataset). A manually curated TF reference list was extracted from the FlyMine database (https://www.flymine.org/flymine). TFs functionally characterized in this work are highlighted.

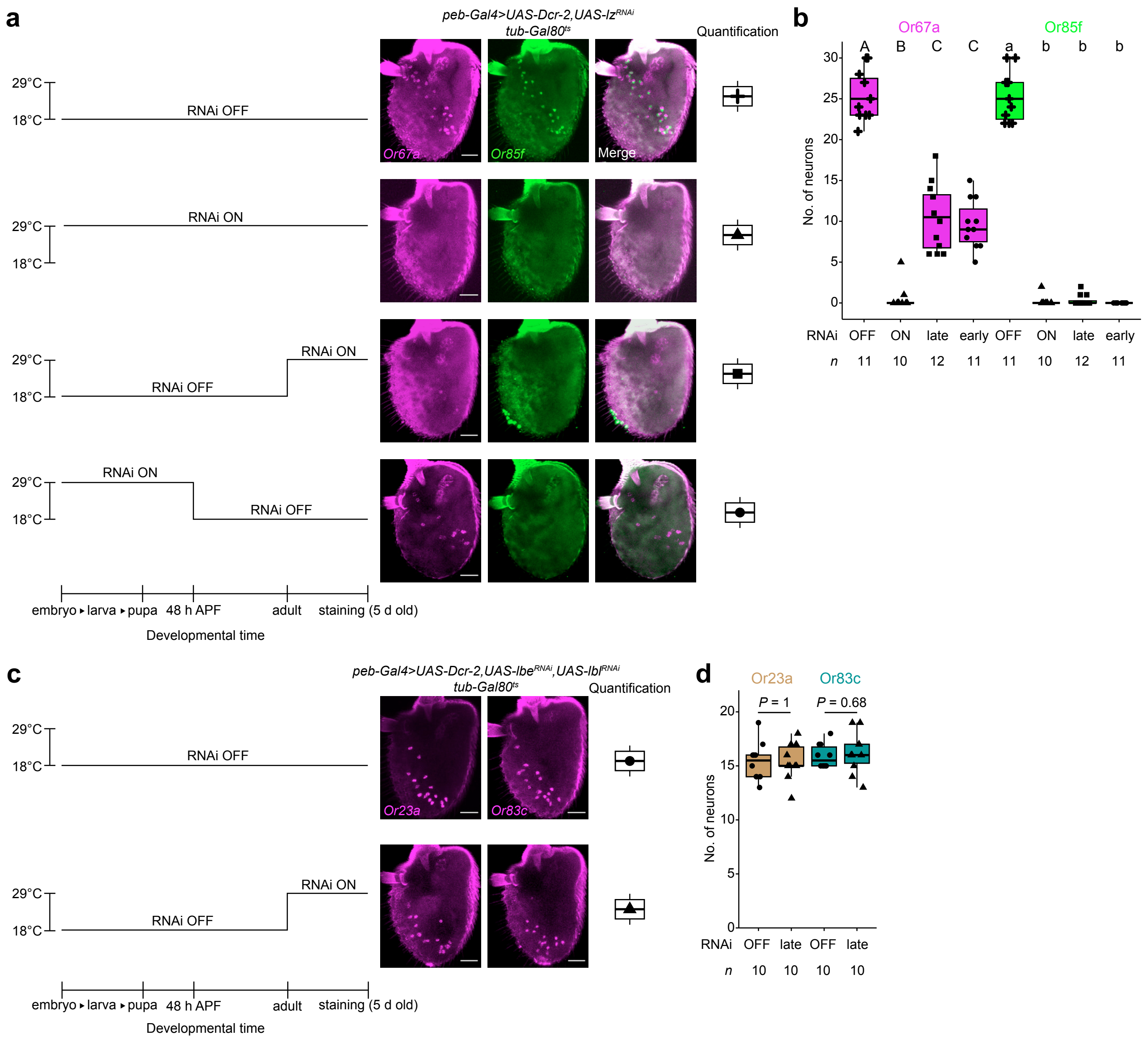

a
peb-Gal4>UAS-Dcr-2,UAS-lz^{RNAi}
tub-Gal80^{ts}
Quantification
29°C
18°C
RNAi OFF
RNAi ON
RNAi ON
RNAi OFF
Or67a
Or85f
Merge
embryo ▸ larva ▸ pupa 48 h APF adult staining (5 d old)
Developmental time
b
Or67a
Or85f
A B C C a b b b
No. of neurons
RNAi OFF ON late early OFF ON late early
n 11 10 12 11 11 10 12 11
c
peb-Gal4>UAS-Dcr-2,UAS-lbe^{RNAi},UAS-lbl^{RNAi}
tub-Gal80^{ts}
Quantification
Or23a
Or83c
d
Or23a
Or83c
P = 1
P = 0.68
No. of neurons
RNAi OFF late OFF late
n 10 10 10 10

Supplementary Fig. 9. Temporal functions of Lz and Lbe/Lbl TFs in OSN development.

a Experimental design and RNA FISH on whole-mount antennae of *peb-Gal4,UAS-Dcr-2/+;UAS-lz*^{RNAi}*/+;tub-Gal80*^{ts}*/+* animals. Scale bars, 25 μm.

b Quantification of experiments in **a**. Letters indicate significant differences; Wilcoxon rank sum test followed by Bonferroni correction for multiple comparisons.

c Experimental design and RNA FISH experiment on whole-mount antennae of *peb-Gal4,UAS-Dcr-2/+;UAS-lbe*^{RNAi}*/+;UAS-lbl*^{RNAi}*/tub-Gal80*^{ts} animals. Scale bars, 25 μm.

d Quantification of experiments in **c**. *P* values are shown (two-sided *t* test).

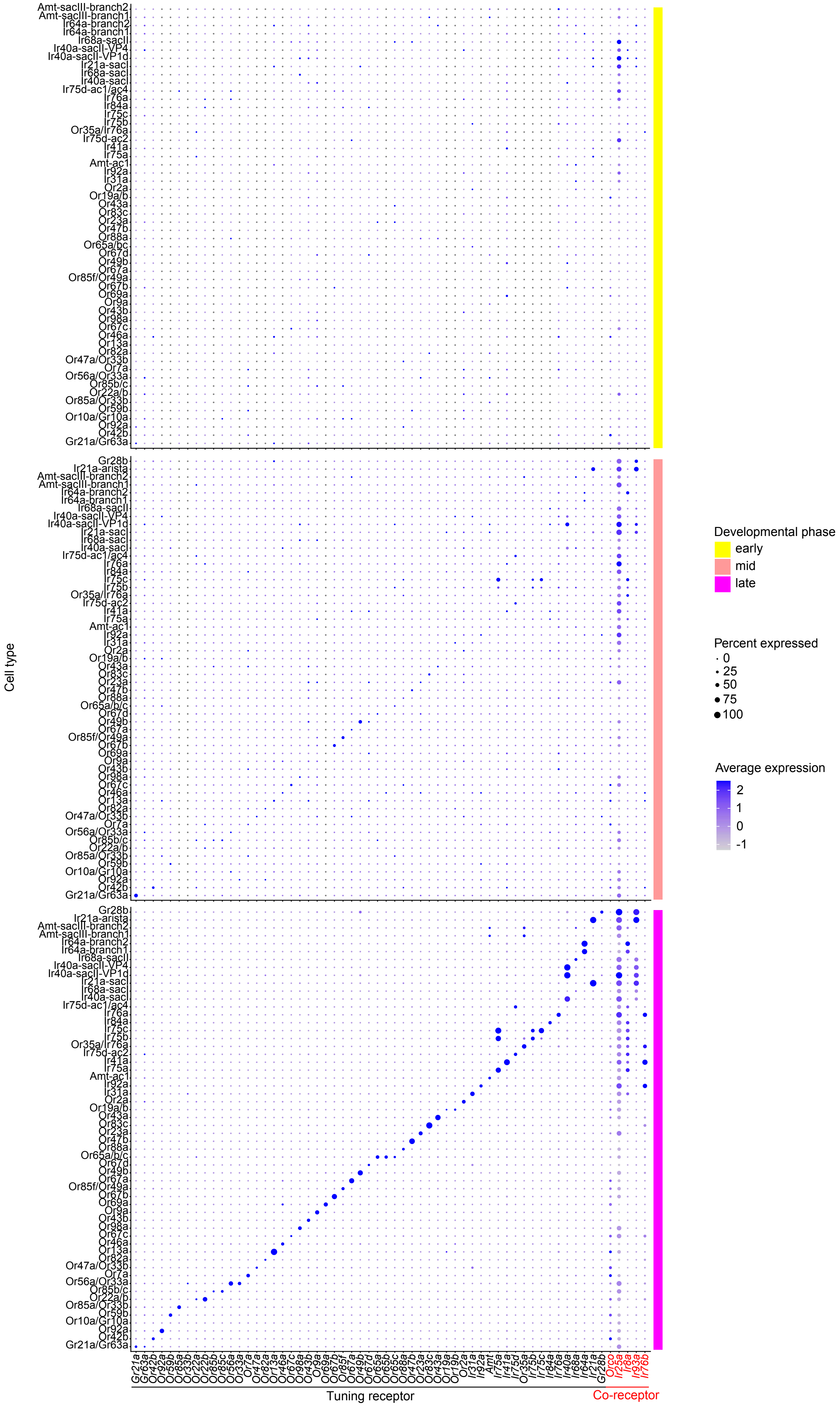
Cell type
Tuning receptor
Co-receptor
Developmental phase
early
mid
late
Percent expressed
0
25
50
75
100
Average expression
2
1
0
-1
Amt-sacIII-branch2
Amt-sacIII-branch1
Ir64a-branch2
Ir64a-branch1
Ir68a-sacII
Ir40a-sacII-VP4
Ir40a-sacII-VP1d
Ir21a-sacI
Ir68a-sacI
Ir40a-sacI
Ir75d-ac1/ac4
Ir76a
Ir84a
Ir75c
Ir75b
Or35a/Ir76a
Ir75d-ac2
Ir41a
Ir75a
Amt-ac1
Ir92a
Ir31a
Or2a
Or19a/b
Or43a
Or83c
Or23a
Or47b
Or88a
Or65a/b/c
Or67d
Or49b
Or67a
Or85f/Or49a
Or67b
Or69a
Or9a
Or43b
Or98a
Or67c
Or46a
Or13a
Or82a
Or47a/Or33b
Or7a
Or56a/Or33a
Or85b/c
Or22a/b
Or85a/Or33b
Or59b
Or10a/Gr10a
Or92a
Or42b
Gr21a/Gr63a
Gr28b
Ir21a-arista
Gr21a
Gr63a
Or42b
Or92a
Or59b
Or85a
Or33b
Or22a
Or22b
Or85b
Or85c
Or56a
Or33a
Or7a
Or47a
Or82a
Or13a
Or46a
Or67c
Or98a
Or43b
Or9a
Or69a
Or67b
Or85f
Or49a
Or67a
Or49b
Or67d
Or65a
Or65b
Or65c
Or88a
Or47b
Or23a
Or83c
Or43a
Or19a
Or19b
Or2a
Ir31a
Ir92a
Amt
Ir75a
Ir41a
Ir75d
Or35a
Ir75b
Ir75c
Ir84a
Ir76a
Ir40a
Ir68a
Ir64a
Ir21a
Gr28b
Orco
Ir25a
Ir8a
Ir93a
Ir76b

Supplementary Fig. 10. Sensory receptor expression during antennal development.

Dot plots illustrating the expression of tuning receptor and co-receptor subunits across sensory neuron types from different developmental phases (control dataset; *peb-Gal4/+;;UAS-unc84:GFP/+*). Only four expected tuning receptors were not detected: (i) *Or49a* (which was, however, detectable in the expected cells – i.e., co-expressed with *Or85f* – in the non-normalized data, (ii) co-expressed *Or10a* and *Gr10a* (likely because the genes have identical annotations, resulting in reads being filtered out due to unintended mapping to two distinct genes), and (iii) *Or33b*, which was not detected in Or85a neurons as reported[4], but instead detected in a very small fraction of Or56a/Or33a neurons**,** potentially reflecting strain specificity. Cells of the Ir21a-arista and Gr28b lineages were not identified in the early developmental phase, so these cell types do not feature in the top dot-plot.

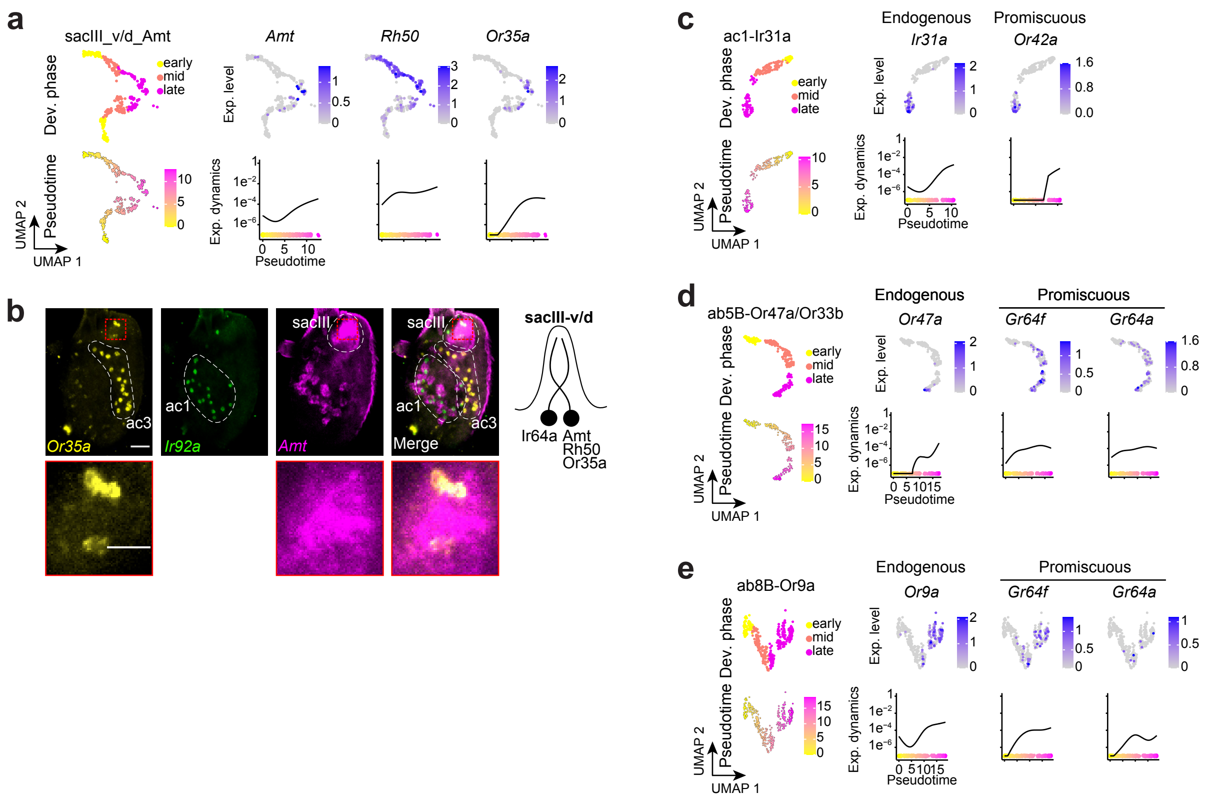
a
sacIII_v/d_Amt
Amt
Rh50
Or35a
Dev. phase
early
mid
late
Exp. level
Pseudotime
Exp. dynamics
UMAP 2
UMAP 1
b
Or35a
Ir92a
Amt
Merge
sacIII
ac1
ac3
sacIII-v/d
Ir64a
Amt
Rh50
Or35a
c
ac1-Ir31a
Endogenous
Ir31a
Promiscuous
Or42a
d
ab5B-Or47a/Or33b
Endogenous
Or47a
Promiscuous
Gr64f
Gr64a
e
ab8B-Or9a
Endogenous
Or9a
Promiscuous
Gr64f
Gr64a

Supplementary Fig. 11. Examples of receptor co-expression.

a Top: UMAPs of the sacIII_v/d-Amt lineage (control dataset) illustrating the developmental phases and receptor expression patterns; *Rh50* encodes an ammonia transporter that is co-expressed with *Amt* in these ammonia-sensing neurons although its role is unclear[5]. Bottom: a pseudotime UMAP and corresponding receptor expression dynamics.

b Left: RNA FISH on whole-mount antennae of control (*peb-Gal4*) animals (*n* = 10-12). The ac3 and ac1 sensilla zones are indicated. Bottom row shows a higher magnification of sacIII-v/d-Amt neurons co-expressing *Amt* and *Or35a* in a confocal Z-slices. Scale bars, 25 μm (top images) or 10 μm (bottom images). Right: schematic of olfactory (co)-receptor subunits expressed in sacIII-v/d-Amt neurons.

c-e Top: UMAPs of the ac1-Ir31a **c**, ab5B-Or47a/Or33b **d** and ab8B-Or9a **e** lineages (control dataset) illustrating the developmental phases and receptor expression patterns. Bottom: pseudotime UMAPs and corresponding receptor expression dynamics.

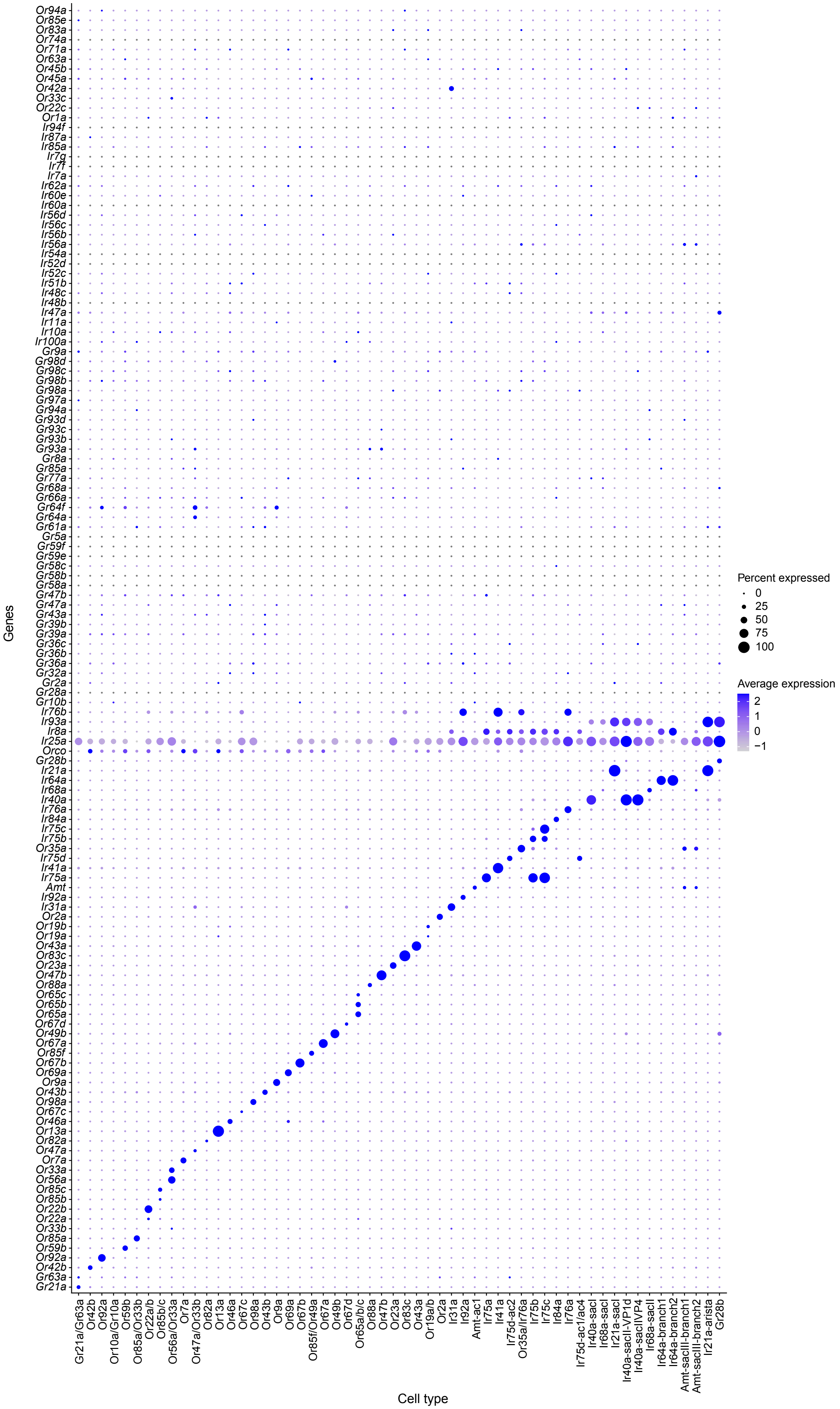

Genes
Or94a
Or85e
Or83a
Or74a
Or71a
Or63a
Or45b
Or45a
Or42a
Or33c
Or22c
Or1a
Ir94f
Ir87a
Ir85a
Ir7g
Ir7f
Ir7a
Ir62a
Ir60e
Ir60a
Ir56d
Ir56c
Ir56b
Ir56a
Ir54a
Ir52d
Ir52c
Ir51b
Ir48c
Ir48b
Ir47a
Ir11a
Ir10a
Ir100a
Gr9a
Gr98d
Gr98c
Gr98b
Gr98a
Gr97a
Gr94a
Gr93d
Gr93c
Gr93b
Gr93a
Gr8a
Gr85a
Gr77a
Gr68a
Gr66a
Gr64f
Gr64a
Gr61a
Gr5a
Gr59f
Gr59e
Gr58c
Gr58b
Gr58a
Gr47b
Gr47a
Gr43a
Gr39b
Gr39a
Gr36c
Gr36b
Gr36a
Gr32a
Gr2a
Gr28a
Gr10b
Ir76b
Ir93a
Ir8a
Ir25a
Orco
Gr28b
Ir21a
Ir64a
Ir68a
Ir40a
Ir76a
Ir84a
Ir75c
Ir75b
Or35a
Ir75d
Ir41a
Ir75a
Amt
Ir92a
Ir31a
Or2a
Or19b
Or19a
Or43a
Or83c
Or23a
Or47b
Or88a
Or65c
Or65b
Or65a
Or67d
Or49b
Or67a
Or85f
Or67b
Or69a
Or9a
Or43b
Or98a
Or67c
Or46a
Or13a
Or82a
Or47a
Or7a
Or33a
Or56a
Or85c
Or85b
Or22b
Or22a
Or33b
Or85a
Or59b
Or92a
Or42b
Gr63a
Gr21a
Gr21a/Gr63a
Or42b
Or92a
Or10a/Gr10a
Or59b
Or85a/Or33b
Or22a/b
Or85b/c
Or56a/Or33a
Or7a
Or47a/Or33b
Or82a
Or13a
Or46a
Or67c
Or98a
Or43b
Or9a
Or69a
Or85f/Or49a
Or67b
Or67a
Or49b
Or67d
Or65a/b/c
Or88a
Or47b
Or23a
Or83c
Or43a
Or19a/b
Or2a
Ir31a
Ir92a
Amt-ac1
Ir75a
Ir41a
Ir75d-ac2
Or35a/Ir76a
Ir75b
Ir75c
Ir84a
Ir76a
Ir75d-ac1/ac4
Ir40a-sacI
Ir68a-sacI
Ir21a-sacI
Ir40a-sacII-VP1d
Ir40a-sacIIVP4
Ir68a-sacII
Ir64a-branch1
Ir64a-branch2
Amt-sacIII-branch1
Amt-sacIII-branch2
Ir21a-arista
Gr28b
Cell type
Percent expressed
0
25
50
75
100
Average expression
2
1
0
−1

Supplementary Fig. 12. Global survey of chemosensory receptor expression in late stages.
Expression of all detected sensory receptor subunits in the late developmental phase (control dataset).

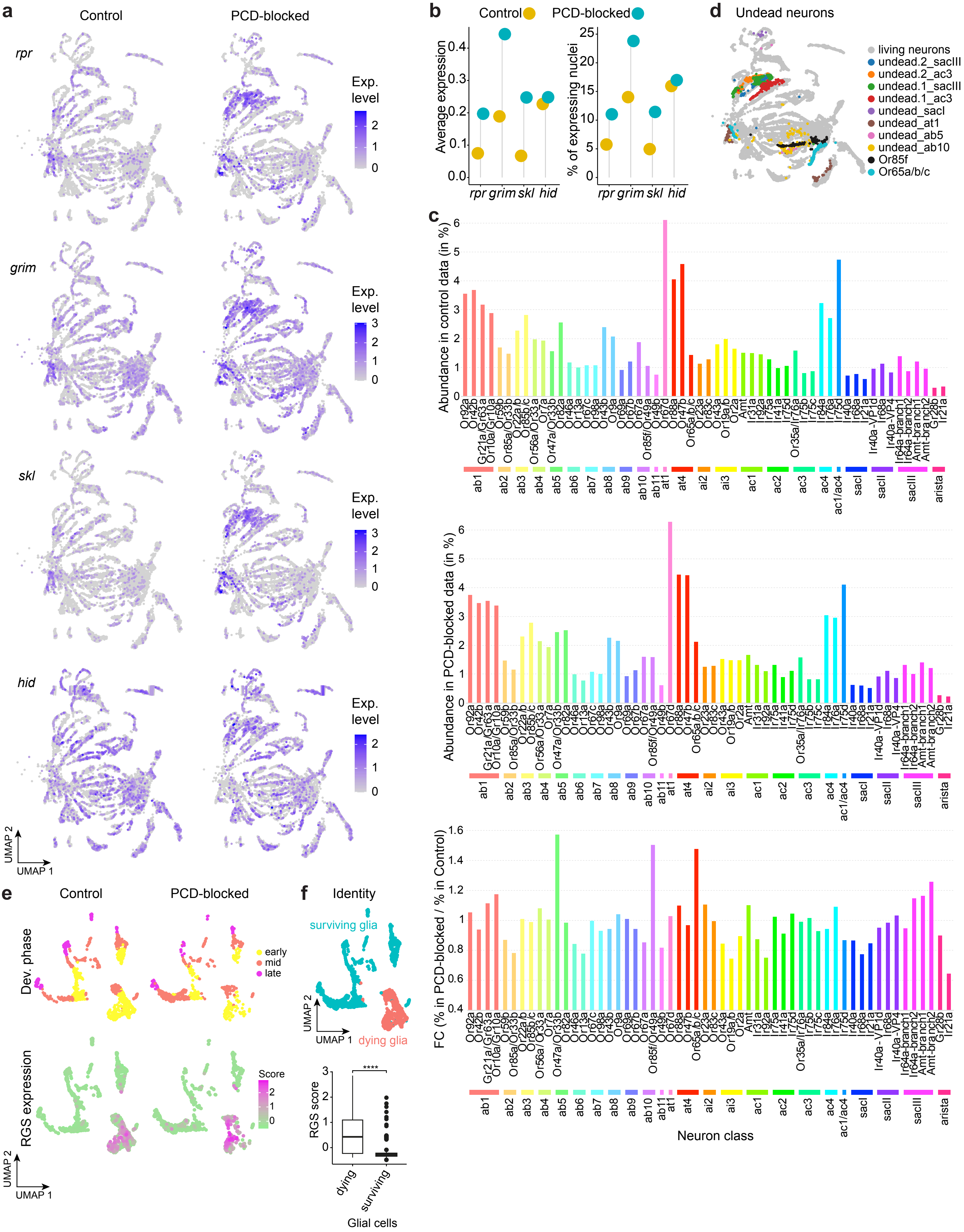
a
Control
PCD-blocked
rpr
grim
skl
hid
Exp. level
UMAP 2
UMAP 1
b
Control
PCD-blocked
Average expression
% of expressing nuclei
rpr grim skl hid
d
Undead neurons
living neurons
undead.2_saclII
undead.2_ac3
undead.1_saclII
undead.1_ac3
undead_sacI
undead_at1
undead_ab5
undead_ab10
Or85f
Or65a/b/c
c
Abundance in control data (in %)
Abundance in PCD-blocked data (in %)
FC (% in PCD-blocked / % in Control)
Neuron class
ab1 ab2 ab3 ab4 ab5 ab6 ab7 ab8 ab9 ab10 ab11 at1 at4 ai2 ai3 ac1 ac2 ac3 ac4 ac1/ac4 sacI sacII sacIII arista
e
Control
PCD-blocked
Dev. phase
early
mid
late
RGS expression
Score
f
Identity
surviving glia
dying glia
RGS score
****
dying
surviving
Glial cells

Supplementary Fig. 13. Expression of pro-apoptotic genes.
a UMAPs illustrating the expression of the pro-apoptotic genes *reaper* (*rpr*), *grim*, *sickle* (*skl*) and *head involution defective* (*hid)* in annotated nuclei of control and PCD-blocked datasets.
b Average expression (left) and fraction of positive nuclei (right) for these genes in control and PCD-blocked datasets.
c Abundance of each neuronal class in control (top) and PCD-blocked (middle) datasets, calculated as the percentage of nuclei for each class relative to the total number of nuclei (from 36 h APF, excluding those forming new clusters in the PCD-blocked dataset) and fold-change in the abundance of each class in the PCD-blocked dataset relative to the control dataset (bottom). Note the Or85b/c population in ab3 includes the small fraction of this cell type that are housed in ab11 as we could not distinguish these two populations, hence ab11 is only represented by Or49b neurons.
d UMAP highlighting all undead neurons able to be annotated in the PCD-blocked dataset.
e UMAPs of subclustered glial cells (from Fig. 1c and e) in control and PCD-blocked datasets illustrating the developmental phases (top) and RGS expression (bottom), revealing cells with a high RGS score.
f Top: glial cells annotation in integrated control and PCD-blocked data. Bottom: RGS score (**** indicates $P < 0.0001$, Wilcoxon rank sum test).

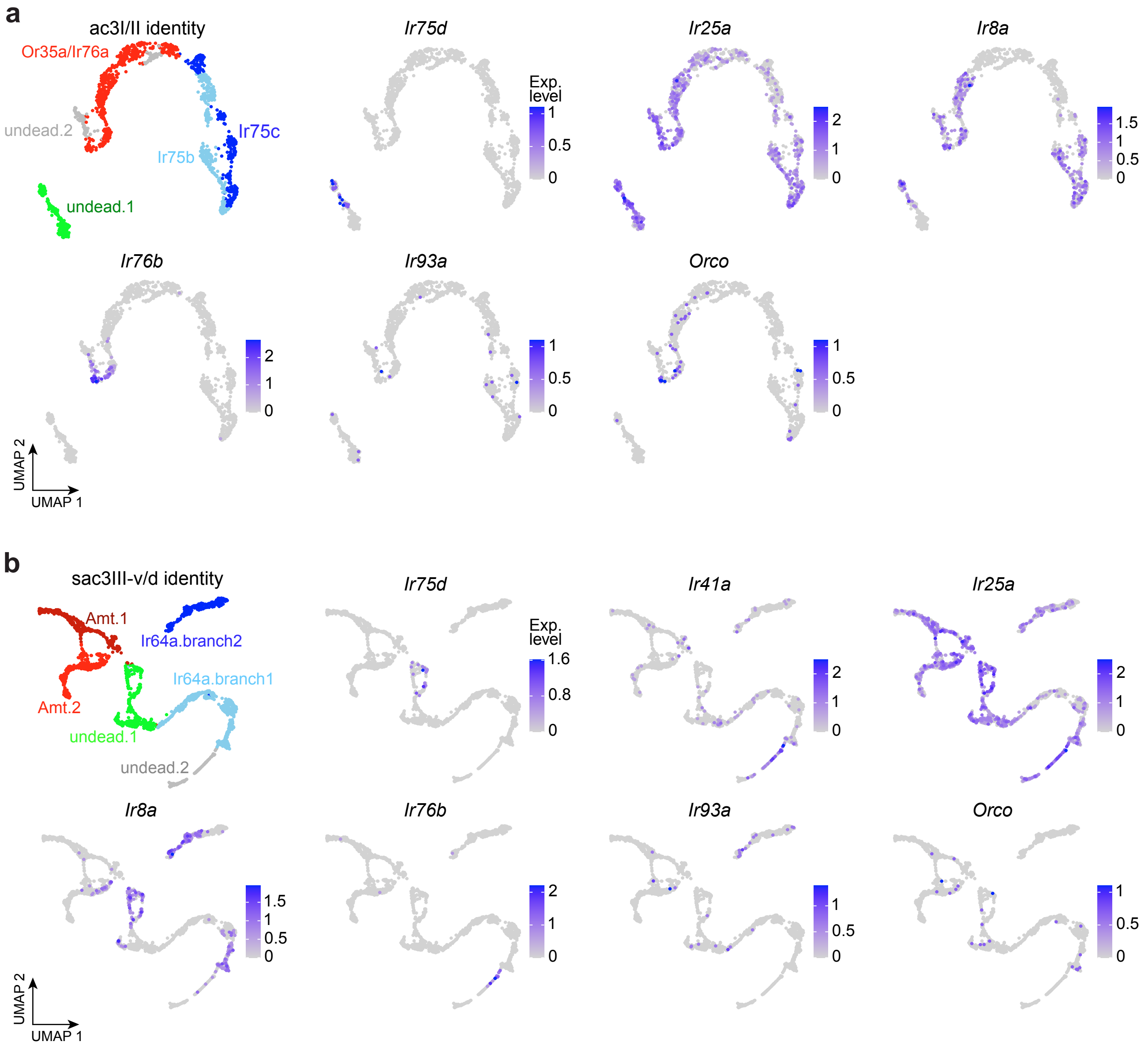
a
ac3I/II identity
Or35a/Ir76a
undead.2
Ir75c
Ir75b
undead.1
Ir75d
Exp. level
1
0.5
0
Ir25a
2
1
0
Ir8a
1.5
1
0.5
0
Ir76b
2
1
0
Ir93a
1
0.5
0
Orco
1
0.5
0
UMAP 2
UMAP 1
b
sac3III-v/d identity
Amt.1
Ir64a.branch2
Amt.2
Ir64a.branch1
undead.1
undead.2
Ir75d
Exp. level
1.6
0.8
0
Ir41a
2
1
0
Ir25a
2
1
0
Ir8a
1.5
1
0.5
0
Ir76b
2
1
0
Ir93a
1
0.5
0
Orco
1
0.5
0
UMAP 2
UMAP 1

Supplementary Fig. 14. Expression of co-receptors in undead neurons.
a UMAPs of the integrated ac3I/II dataset illustrating the expression of tuning receptors (as in Fig. 5d) and co-receptors in normal and undead neurons.
b UMAPs of the integrated sacIII-v/d dataset illustrating the expression of tuning receptors (as in Fig. 5k) and co-receptors in normal and undead neurons.

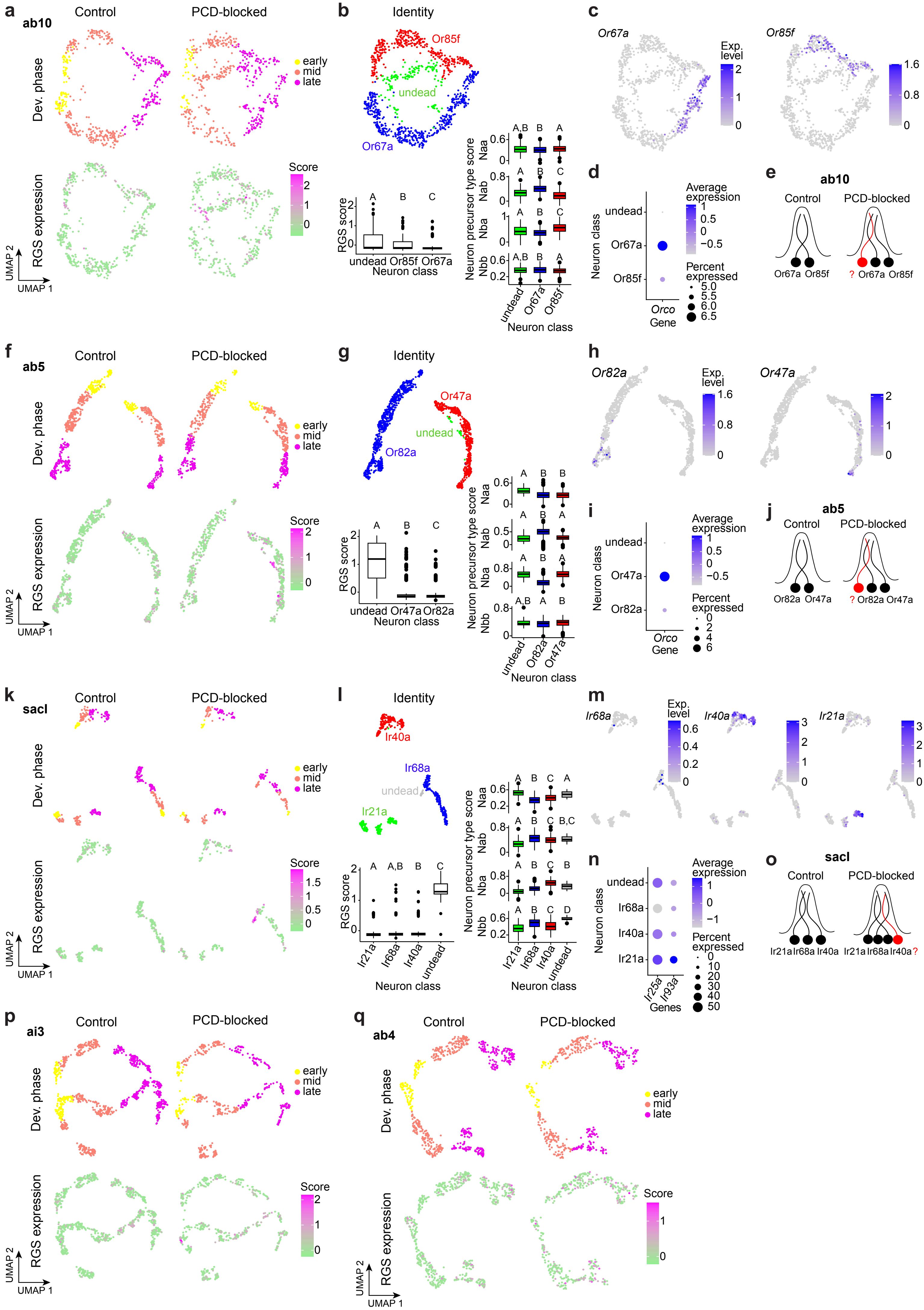

a
ab10
Control
PCD-blocked
Dev. phase
early
mid
late
RGS expression
Score
UMAP 2
UMAP 1
b
Identity
Or85f
undead
Or67a
RGS score
Neuron class
Neuron precursor type score
Naa
Nab
Nba
Nbb
c
Or67a
Or85f
Exp. level
d
Neuron class
Average expression
Percent expressed
Orco
Gene
e
ab10
Control
PCD-blocked
?
f
ab5
g
Or47a
Or82a
h
i
j
ab5
k
sacI
l
Ir40a
Ir68a
Ir21a
m
n
Ir25a
Ir93a
Genes
o
sacI
p
ai3
q
ab4

Supplementary Fig. 15. Analysis of undead neuron lineages in additional sensilla.

a UMAPs of the ab10 lineage from control and PCD-blocked datasets illustrating the developmental phases (top) and RGS expression (bottom), revealing that some cells with high RGS score are present exclusively in PCD-blocked animals.

b Lineage annotation of the ab10 sensillum in control and PCD-blocked integrated datasets (top), ranked RGS score (left-to-right) (bottom left) and precursor type score (bottom right) for each OSN type in the integrated datasets. Boxes show the median (thick line), first and third quartiles, while whiskers indicate data distribution limits, here and elsewhere.

c Expression of the indicated receptors in the integrated control and PCD-blocked datasets. No receptor was robustly detected in the undead neuron population.

d Expression of *Orco* in the integrated control and PCD-blocked datasets.

e Inferred states of the ab10 sensillum in control and PCD-blocked antennae.

f As in **a** for the ab5 lineage.

g As in **b** for the ab5 lineage.

h Expression of the indicated receptors in the integrated control and PCD-blocked datasets. No receptor was robustly detected in the undead neuron population.

i Expression of *Orco* in the integrated control and PCD-blocked datasets.

j Inferred states of the ab5 sensillum in control and PCD-blocked antennae.

k As in **a** for the sacI lineage.

l As in **b** for the sacI lineage.

m Expression of the indicated receptors in the integrated control and PCD-blocked datasets. No receptor was robustly detected in the undead neuron population.

n Expression of *Ir25a* and *Ir93a* in the integrated control and PCD-blocked datasets. Note that *Ir8a* and *Ir76b* were not detected in these cells.

o Inferred states of the sacI sensillum in control and PCD-blocked antennae.

p-q UMAPs of the ai3 (**p**) and ab4 (**q**) lineages from control and PCD-blocked datasets illustrating the developmental phases (top) and RGS expression (bottom). No undead neurons were apparent.

b,g,l Letters indicate significant differences: $P < 0.05$ in pairwise comparisons (Wilcoxon rank sum test followed by Bonferroni correction for multiple comparisons).

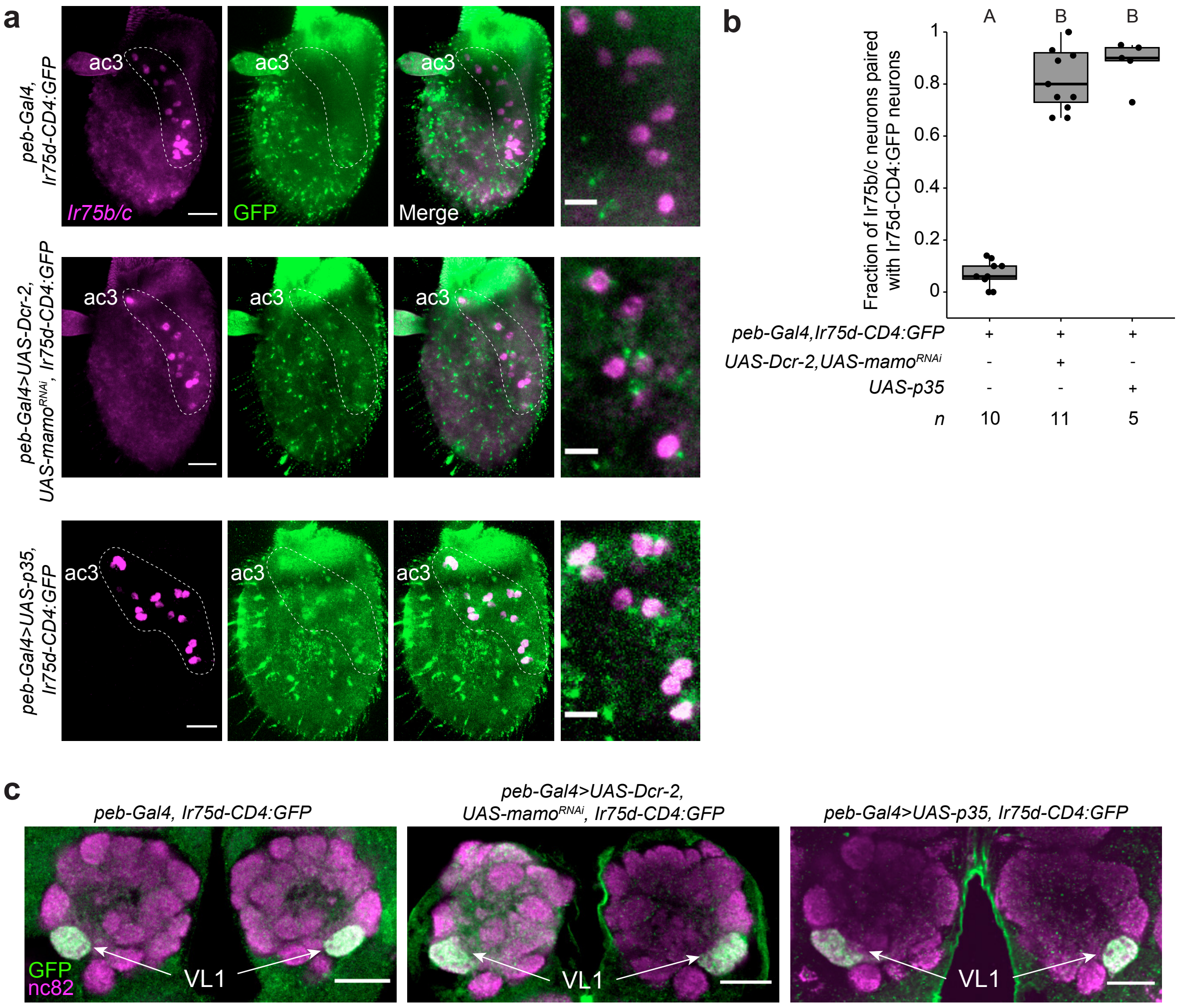

a
peb-Gal4, Ir75d-CD4:GFP
peb-Gal4>UAS-Dcr-2, UAS-mamoRNAi, Ir75d-CD4:GFP
peb-Gal4>UAS-p35, Ir75d-CD4:GFP
ac3
Ir75b/c
GFP
Merge
b
Fraction of Ir75b/c neurons paired with Ir75d-CD4:GFP neurons
A
B
B
1
0.8
0.6
0.4
0.2
0
peb-Gal4,Ir75d-CD4:GFP + + +
UAS-Dcr-2,UAS-mamoRNAi - + -
UAS-p35 - - +
n 10 11 5
c
peb-Gal4, Ir75d-CD4:GFP
peb-Gal4>UAS-Dcr-2, UAS-mamoRNAi, Ir75d-CD4:GFP
peb-Gal4>UAS-p35, Ir75d-CD4:GFP
GFP
nc82
VL1

Supplementary Fig. 16. Analysis of Ir75d neuron projection patterns.

a *Ir75b/c* RNA FISH and anti-GFP immunofluorescence on whole-mount antennae of control (*peb-Gal4/+;Ir75d-CD4:GFP/+*), *mamo*^{RNAi} (*peb-Gal4,UAS-Dcr-2/+;UAS-mamo*^{RNAi}*/Ir75d-CD4:GFP*) and PCD-blocked (*peb-Gal4/+;UAS-p35/Ir75d-CD4:GFP*) animals. Right column images show a higher magnification of 5 adjacent confocal Z-slices in the ac3 zone. Scale bars, 25 µm (or 10 µm in right column images).

b Quantification from **a** of the fraction of Ir75b/c neurons paired with Ir75d-CD4:GFP neurons. Letters indicate significant differences: $P < 0.05$ in pairwise comparisons (Wilcoxon rank sum test followed by Bonferroni correction for multiple comparisons). Note that the small fraction of pairs detected as such in control condition reflects the close proximity of some Ir75d-ac2 neurons with Ir75b/c-ac3 neurons (but not real co-housing within the same sensilla).

c GFP and nc82 immunofluorescence on whole-mount brains of control (*peb-Gal4/+;Ir75d-CD4:GFP/+*) (left), *mamo*^{RNAi} (*peb-Gal4,UAS-Dcr-2/+;UAS-mamo*^{RNAi}*/Ir75d-CD4:GFP*) (middle) and PCD-blocked (*peb-Gal4/+;UAS-p35/Ir75d-CD4:GFP*) (right) animals (*n* = 4, 8 and 6, respectively). Scale bars, 25 µm.

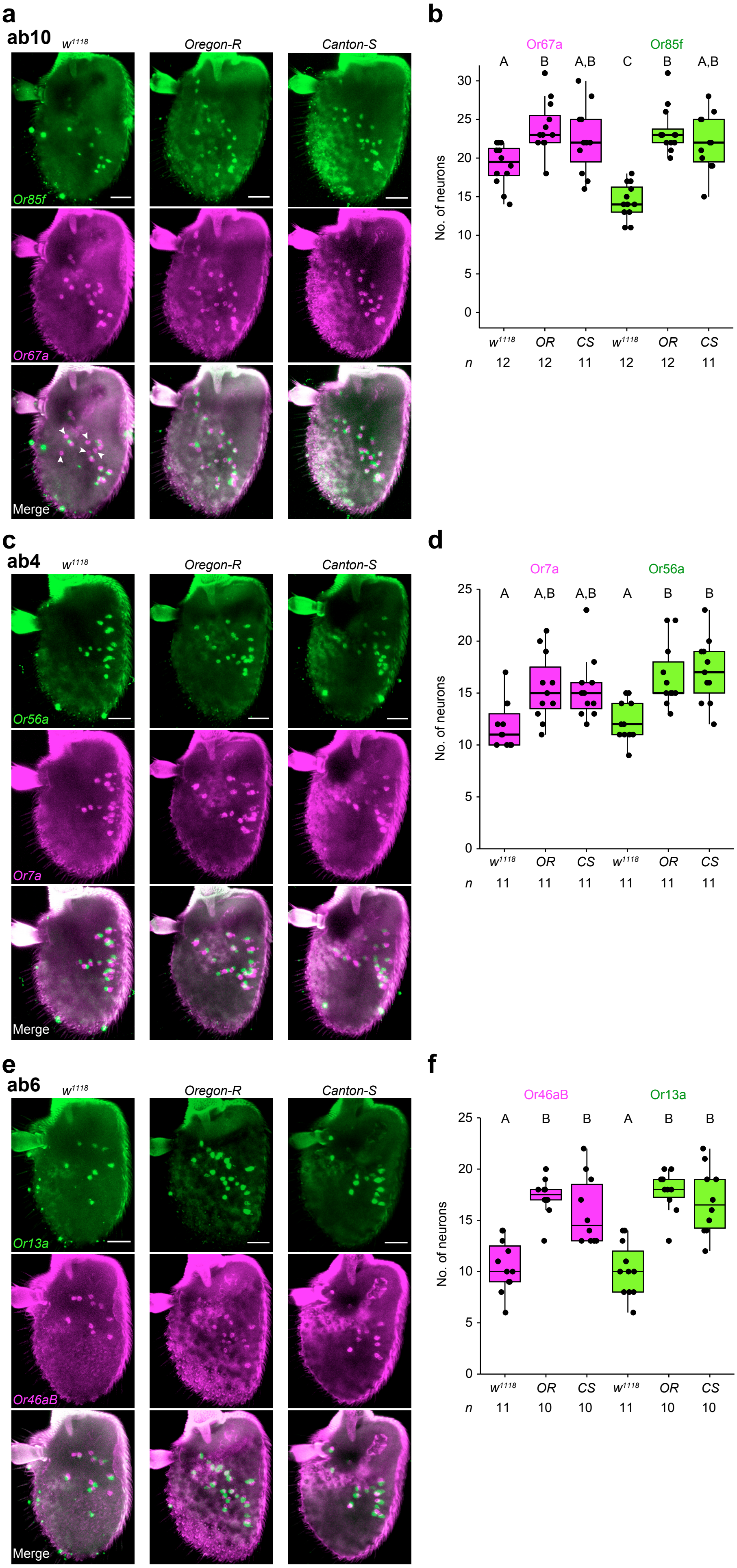
a
ab10
w1118
Oregon-R
Canton-S
Or85f
Or67a
Merge
b
Or67a
Or85f
A B A,B C B A,B
No. of neurons
0 5 10 15 20 25 30
w1118 OR CS w1118 OR CS
n 12 12 11 12 12 11
c
ab4
w1118
Oregon-R
Canton-S
Or56a
Or7a
Merge
d
Or7a
Or56a
A A,B A,B A B B
No. of neurons
0 5 10 15 20 25
w1118 OR CS w1118 OR CS
n 11 11 11 11 11 11
e
ab6
w1118
Oregon-R
Canton-S
Or13a
Or46aB
Merge
f
Or46aB
Or13a
A B B A B B
No. of neurons
0 5 10 15 20 25
w1118 OR CS w1118 OR CS
n 11 10 10 11 10 10

Supplementary Fig. 17. Intraspecific variation in ab10 neuron numbers.
a RNA FISH on whole-mount antennae of w^{1118}, *Oregon-R* and *Canton-S* animals. Arrowheads indicate Or67a neurons unpaired with Or85f neurons. Scale bar, 25 µm.
b Quantification of neurons from **a**.
c RNA FISH on whole-mount antennae of w^{1118}, *Oregon-R* and *Canton-S* animals. Scale bar, 25 µm.
d Quantification of neurons from **c**.
e RNA FISH on whole-mount antennae of w^{1118}, *Oregon-R* and *Canton-S* animals. Scale bar, 25 µm.
f Quantification of neurons from **e**.
b,d,f Letters indicate significant differences: $P < 0.05$ in pairwise comparisons (Wilcoxon rank sum test followed by Bonferroni correction for multiple comparisons).

Supplementary Table 1. *Drosophila* strains.

Genotype	Source/reference
w^{1118}	
Canton-S	
Oregon-R	
peb-Gal4	RRID:BDSC_80570
UAS-unc84:GFP	RRID:BDSC_602372
UAS-p35	RRID:BDSC_5072
UAS-Dcr-2	VDRC_60010
UAS-mamo^{RNAi}	VDRC_110310
UAS-lz^{RNAi}	VDRC_330539
UAS-lbe^{RNAi}	VDRC_102377
UAS-lbl^{RNAi}	VDRC_52323
UAS-slp2^{RNAi}	VDRC_37657
UAS-Orco	[6]
UAS-Ir8a	[7]
Or67d-Gal4	[8]
Ir84a-Gal4	[9]
at1-Gal4 (GMR82D08-Gal4)	[10]
tub-Gal80^{ts}	RRID:BDSC_7018
UAS-slp2-ORF-3HA	FlyORF
Ir75d-CD4:GFP	*This work*

Supplementary Table 2. RNA FISH probes.

Gene	Reference/source	Primer sequences (5'-3' Fwd / Rev) (standard FISH) or target sequence (HCR-FISH)
Amt	Molecular Instruments	NM_001104330.3
Ir31a	Molecular Instruments	NM_164926.2
Ir41a	Molecular Instruments	NM_206022.4
Ir64a	Molecular Instruments	NM_139705.1
Ir75b	Molecular Instruments	KY205710.1
Ir75d	Molecular Instruments	NM_140817.3
Ir76a	Molecular Instruments	NM_001104177.3
Ir84a	Molecular Instruments	NM_141463.2
Ir92a	Molecular Instruments	NM_001104375.2
lbe	Molecular Instruments	NM_079711.3
lbl	Molecular Instruments	NM_079710.4
lz	Molecular Instruments	NM_078544.3
Or7a	Molecular Instruments	NM_078526.1
Or13a	Molecular Instruments	NM_078635.3
Or19a	Molecular Instruments	NM_080274.3
Or23a	Molecular Instruments	NM_078734.4
Or35a	Molecular Instruments	NM_165117.2
Or43a	Molecular Instruments	NM_078923.3
Or46aB	Molecular Instruments	NM_206071.2

Or47b	Molecular Instruments	NM_078966.3
Or65a	Molecular Instruments	NM_168163.1
Or56a	Molecular Instruments	NM_079072.2
Or67a	Molecular Instruments	NM_079281.4
Or67b	Molecular Instruments	NM_079283.5
Or67d	Molecular Instruments	NM_140133.2
Or69a	Molecular Instruments	NM_206348.1
Or83c	Molecular Instruments	NM_079520.3
Or85f	Molecular Instruments	NM_079565.3
Or88a	Molecular Instruments	NM_079624.3
Ir75b		CCGCATCTATGTGGAAACCT / CAATATGCCCATGCAGAGAA
Or35a		TAGCTGTGCGATGTCTTG / GTCAAAAGCAGTGGCACAAA
Ir75d		GACAGGATCTGGAGGGCATA / ACACCCACACATCGTTCTCA

Supplementary Table 3. Antibodies.

**Antibody**	**Dilution**	**Reference/source**	**Identifier**
Chicken anti-GFP	1:500	Abcam	ab13970
Mouse anti-Bruchpilot (nc82)	1:10	DSHB	nc82
anti-DIG-POD	1:300	Roche Diagnostics AG	11 207 733 910
anti-Fluorescein-POD	1:300	Roche Diagnostics AG	11 426 346 910
Alexa Fluor 488 Goat anti-Chicken	1:500	Abcam	ab150169
Cy5 Goat anti-Mouse	1:250	Jackson Immunoresearch	115-175-166

Supplementary Table 4. Odors.

**Odor**	**CAS**	**Source**
11-cis-vaccenyl acetate (*c*VA)	6186-98-7	Pherobank
E2-hexenal	6728-26-3	Sigma-Aldrich
1-hexanol	111-27-3	Acros Organics
hexyl acetate	142-92-7	Sigma-Aldrich
1-octanol	111-87-5	Acros Organics
2-oxopentanoic acid	1821-02-9	Sigma-Aldrich
paraffin oil (solvent)	8012-95-1	Acros Organics
phenethylamine	64-04-0	Acros Organics
phenylacetaldehyde	122-78-1	Alfa Aesar

Supplementary References

1. Li H*, et al.* Fly Cell Atlas: A single-nucleus transcriptomic atlas of the adult fruit fly. *Science.* **375**, eabk2432 (2022).

2. McLaughlin CN*, et al.* Single-cell transcriptomes of developing and adult olfactory receptor neurons in *Drosophila*. *Elife.* **10**, e63856 (2021).

3. Scalzotto M*, et al.* Pheromone sensing in *Drosophila* requires support cell-expressed Osiris 8. *BMC Biol.* **20**, 230 (2022).

4. Fishilevich E, Vosshall LB. Genetic and functional subdivision of the *Drosophila* antennal lobe. *Curr Biol.* **15**, 1548-1553 (2005).

5. Vulpe A*, et al.* An ammonium transporter is a non-canonical olfactory receptor for ammonia. *Curr Biol.* **31**, 3382-3390 e3387 (2021).

6. Benton R, Sachse S, Michnick SW, Vosshall LB. Atypical membrane topology and heteromeric function of *Drosophila* odorant receptors *in vivo*. *PLOS Biol.* **4**, e20 (2006).

7. Abuin L, Bargeton B, Ulbrich MH, Isacoff EY, Kellenberger S, Benton R. Functional architecture of olfactory ionotropic glutamate receptors. *Neuron.* **69**, 44-60 (2011).

8. Kurtovic A, Widmer A, Dickson BJ. A single class of olfactory neurons mediates behavioural responses to a *Drosophila* sex pheromone. *Nature.* **446**, 542-546 (2007).

9. Grosjean Y*, et al.* An olfactory receptor for food-derived odours promotes male courtship in *Drosophila*. *Nature.* **478**, 236-240 (2011).

10. Chai PC, Cruchet S, Wigger L, Benton R. Sensory neuron lineage mapping and manipulation in the *Drosophila* olfactory system. *Nat Commun.* **10**, 643 (2019).
