# Supplementary material for: Multilayer regulation underlies the functional precision and evolutionary potential of the olfactory system
Source: Nat Commun. 2025 Oct 28;16:9514. doi: 10.1038/s41467-025-64514-8 (PMC12569201; doi:10.1038/s41467-025-64514-8)
Supplement: Supplementary file 2 — Description of Additional Supplementary Files [file 41467_2025_64514_MOESM2_ESM.pdf]

Description of Additional Supplementary Files

Supplementary Data 1: Neuron precursor type marker genes.

Supplementary Data 2: Top marker genes in undead and normal Ir75d neurons, and undead and Or67d at1 sensilla neurons.

Supplementary Data 3: RNAi screen data.
